# Toxin-antitoxin system gene mutations driving *Mycobacterium tuberculosis* transmission revealed by whole genome sequencing

**DOI:** 10.3389/fmicb.2024.1398886

**Published:** 2024-07-31

**Authors:** Yawei Hou, Yifan Li, Ningning Tao, Xianglong Kong, Yameng Li, Yao Liu, Huaichen Li, Zhenguo Wang

**Affiliations:** ^1^Institute of Chinese Medical Literature and Culture, Shandong University of Traditional Chinese Medicine, Jinan, Shandong, China; ^2^Department of Respiratory and Critical Care Medicine, The Third Affiliated Hospital of Shandong First Medical University (Affiliated Hospital of Shandong Academy of Medical Sciences), Jinan, Shandong, China; ^3^Department of Respiratory and Critical Care Medicine, Shandong Provincial Hospital Affiliated to Shandong University, Shandong Provincial Hospital Affiliated to Shandong First Medical University, Jinan, Shandong, China; ^4^Artificial Intelligence Institute Qilu University of Technology (Shandong Academy of Sciences), Jinan, Shandong, China; ^5^The First Clinical Medical College, Shandong University of Traditional Chinese Medicine, Jinan, Shandong, China

**Keywords:** toxin-antitoxin system, *Mycobacterium tuberculosis*, transmission, whole genome sequencing, single nucleotide polymorphisms

## Abstract

**Background:**

The toxin-antitoxin (TA) system plays a vital role in the virulence and pathogenicity of *Mycobacterium tuberculosis* (*M. tuberculosis*). However, the regulatory mechanisms and the impact of gene mutations on *M. tuberculosis* transmission remain poorly understood.

**Objective:**

To investigate the influence of gene mutations in the toxin-antitoxin system on *M. tuberculosis* transmission dynamics.

**Method:**

We performed whole-genome sequencing on the analyzed strains of *M. tuberculosis*. The genes associated with the toxin-antitoxin system were obtained from the National Center for Biotechnology Information (NCBI) Gene database. Mutations correlating with enhanced transmission within the genes were identified by using random forest, gradient boosting decision tree, and generalized linear mixed models.

**Results:**

A total of 13,518 *M. tuberculosis* isolates were analyzed, with 42.29% (*n* = 5,717) found to be part of genomic clusters. Lineage 4 accounted for the majority of isolates (*n* = 6488, 48%), followed by lineage 2 (*n* = 5133, 37.97%). 23 single nucleotide polymorphisms (SNPs) showed a positive correlation with clustering, including *vapB1* G34A, *vapB24* A76C, *vapB2* T171C, *mazF2* C85T, *mazE2* G104A, *vapB31* T112C, *relB* T226A, *vapB11* C54T, *mazE5* T344C, *vapB14* A29G, *parE1* (C103T, C88T), and *parD1* C134T. Six SNPs, including *vapB6* A29C, *vapB31* T112C, *parD1* C134T, *vapB37* G205C, *Rv2653c* A80C, and *vapB2*2 C167T, were associated with transmission clades across different countries. Notably, our findings highlighted the positive association of *vapB6* A29C, *vapB31* T112C, *parD1* C134T, *vapB37* G205C, *vapB1*9 C188T, and *Rv2653c* A80C with transmission clades across diverse regions. Furthermore, our analysis identified 32 SNPs that exhibited significant associations with clade size.

**Conclusion:**

Our study presents potential associations between mutations in genes related to the toxin-antitoxin system and the transmission dynamics of *M. tuberculosis*. However, it is important to acknowledge the presence of confounding factors and limitations in our study. Further research is required to establish causation and assess the functional significance of these mutations. These findings provide a foundation for future investigations and the formulation of strategies aimed at controlling TB transmission.

## 1 Introduction

Tuberculosis (TB) is a global health threat caused by the highly successful human pathogen *Mycobacterium tuberculosis* (*M. tuberculosis*). According to a report by the World Health Organization (WHO), an estimated 10.6 million new TB cases occurred worldwide in 2022, resulting in over 1.3 million deaths (World Health Organization, 2023). Despite the substantial global burden of TB, our knowledge regarding the factors influencing its transmission remains limited. Therefore, it is imperative to delve deeper into the mechanisms underlying the spread of *M. tuberculosis*.

The toxin-antitoxin (TA) system plays a critical biological role in *M. tuberculosis*. Composed of toxins and antitoxins, this system forms a small genetic unit that is widely present in prokaryotes (Schuster and Bertram, 2013; Dai et al., 2022). TA systems have been shown to assist cells in stress adaptation, antibiotic resistance, biofilm formation, persisters, and disease development. Toxins are typically translated into proteins, while antitoxins can be either proteins or RNA (Ogura and Hiraga, 1983; Aizenman et al., 1996; Magnuson, 2007; Fineran et al., 2009; Wang and Wood, 2011; Lobato-Márquez et al., 2016). Based on the nature of antitoxins and the mechanisms which inhibit toxin activity, TA modules can be classified into six distinct types (Page and Peti, 2016). Among these types, type II TA systems are well-characterized, where antitoxins directly interact with toxins to neutralize their effects. Bioinformatics and phylogenetic analyses have revealed the presence of numerous TA systems encoded in the *M. tuberculosis* genome. The retention of these TA systems in members of the *M. tuberculosis* complex suggests their crucial role in regulating metabolic pathways essential for bacterial pathogenesis. Type II TA systems predominate in *M. tuberculosis*. The abundance of TA loci in the *M. tuberculosis* genome raises important questions about their functional diversity (Ramage et al., 2009; Tandon et al., 2019). Previous studies have extensively investigated the various functions of TA systems in *M. tuberculosis* and their potential impact on pathogenic mechanisms (Schippers et al., 2005; Guo et al., 2016). These systems are believed to play a key role in *M. tuberculosis* ‘s response to stressors such as nutrient starvation and antibiotic treatment, promoting its survival and drug resistance (Kim et al., 2018). Additionally, TA systems are associated with the formation of persistent cells, a subpopulation exhibiting drug tolerance that plays a crucial role in establishing chronic infections in *M. tuberculosis* (Merfa et al., 2016). While the importance of toxin-antitoxin systems in *M. tuberculosis* has been acknowledged, our understanding of their specific mechanisms and functions within this bacterium remains limited. Therefore, comprehensive research is required to explore the roles of TA systems and gain deeper insights into the complex biology of *M. tuberculosis*.

Driven by the need to better understand the mechanisms underlying *M. tuberculosis* transmission, we conducted an extensive study investigating the impact of mutations in TA system genes on its spread. Our research aims to elucidate how genetic variations within this system can influence *M. tuberculosis* strain transmission dynamics. Utilizing whole-genome sequencing (WGS), we analyzed the genetic variations present in *M. tuberculosis* isolates at a high-resolution level. This enabled us to identify specific mutations within the TA system genes that may be associated with *M. tuberculosis* transmission. Advanced statistical and bioinformatics techniques, including random forest, gradient boosting decision tree, and generalized linear mixed models, were employed for comprehensive analyses to identify key genetic variants linked to transmission dynamics. We acknowledge challenges posed by confounding factors and population dynamics in our analysis. Future research should incorporate social networks and regional interactions for a more comprehensive understanding. Limitations of our study include a focus on gene analysis, potentially overlooking other important genetic influences such as drug resistance mutations or virulence determinants. Therefore, more comprehensive studies are needed to address these limitations adequately. Our study has yielded significant results, identifying multiple single nucleotide polymorphisms (SNPs) within the toxin-antitoxin system genes that positively correlate with clustering, suggesting their potential role in *M. tuberculosis* transmission. Furthermore, some of these SNPs were found to be associated with transmission clades across different geographical regions, indicating their potential global impact on the spread of *M. tuberculosis*. These findings provide valuable insights into the transmission dynamics of this pathogen and contribute to a more thorough understanding of *M. tuberculosis* transmission.

## 2 Materials and methods

### 2.1 Sample collection

We collected a total of 1,550 samples from patients with culture-positive pulmonary tuberculosis at two medical institutions in China: the Shandong Public Health Clinical Research Center (SPHCC) and Weifang Respiratory Disease Hospital (WRDH). These samples were obtained through analysis of sputum specimens. The sample collection spanned the period from 2011 to 2018. It is important to note that all samples were collected anonymously, and therefore, informed consent was not required as per the approved research protocol. Our study received ethical approval from the Ethics Committee of Shandong Provincial Hospital, which is affiliated with Shandong First Medical University (No.2017-337). This approval ensures that our research adheres to ethical guidelines and safeguards the rights and privacy of the participants involved in the study.

### 2.2 DNA extraction and sequencing

Genomic deoxyribonucleic acid (DNA) was successfully extracted from 1,468 of the 1,550 Shandong *M. tuberculosis* isolates. Gene sequencing was performed at the Beijing Genomic Institute. The genomic DNA was sequenced using an Illumina HiSeq 4,000 system. The resulting sequence data were deposited in the National Center for Biotechnology Information (NCBI) BioProject PRJNA1002108. Quality control of the sequence reads was conducted using Fast QC software, and a total of 1,447 samples passed the quality control criteria. Low-quality raw reads with a sequencing base ≤20 or sequencing fragments length ≤ 20 were excluded from the paired-end sequencing process. During the analysis, two isolates were accidentally lost, resulting in 1445 isolates being included for further analysis. The reads of these 1,445 strains, along with 12,132 *M. tuberculosis* isolates downloaded from previous studies and collected from 52 countries and 18 regions worldwide, were aligned to the H37Rv reference genome (NC_000962.3) using BWA-MEM (version 0.7.17-r1188) (Luo et al., 2015; Yang et al., 2017; Coll et al., 2018; Hicks et al., 2018; Koster et al., 2018; Liu et al., 2018; Chen et al., 2019; Huang et al., 2019; Jiang et al., 2020). To improve the alignment quality, clipped alignments and duplicated reads were removed using samclip (v0.4.0) and samtools markdup (v1.15), respectively. Samples with a coverage rate below 98% or a depth less than 20× were excluded from the analysis (Jajou et al., 2019; Yang et al., 2021). Additionally, 55 *Mycobacterium bovis* isolates, one *Mycobacterium caprae* isolate, and three *Mycobacterium orygis* isolates were also excluded. In summary, a total of 13,518 genomes were analyzed in this study. Specific sample numbers can be found in Supplementary Tables 1, 2.

### 2.3 Single nucleotide polymorphism (SNP) analysis

After performing variant calling, we proceeded with additional filtering steps to enhance the quality of the detected variants. This involved employing Free Bayes (version 1.3.2) with an included filter parameter “FMT/GT = “1/1″ && QUAL> = 100 && FMT/DP > = 10 && (FMT/AO)/(FMT/DP) > = 0.” and Bcftools (version 1.15.1) for further refinement of the identified variants. To ensure the accuracy of our analysis, we excluded SNPs located within repetitive regions. This includes polymorphic sequences rich in GC found in PE/PPE genes, directly repeated SNPs, and repetitive bases identified using Tandem Repeat Finder (version 4.09) and RepeatMask (version 4.1.2-P1) (Li et al., 2009; Liu et al., 2019). The annotation for each candidate SNP was determined using SnpEff, version 4.11. The resulting output was obtained by utilizing the Python programming language (Cingolani et al., 2012).

### 2.4 Phylogenetic analysis

Phylogenetic lineages were inferred based on specific SNPs following the methodology described by Coll et al. (2014) (Supplementary Tables 1, 2). Maximum-likelihood phylogenetic and phylogenomic analyses were conducted using IQ-TREE version 1.6.12. The phylogeny was constructed using the general time reversible (GTR) model of nucleotide substitution with the GAMMA model of rate heterogeneity, and bootstrap replicates were performed with 100 iterations. To establish the phylogenetic relationships, the genome of the *Mycobacterium canettii* strain CIPT 140010059 (NC_15848.1) was used as an outgroup (Nguyen et al., 2015). The resulting phylogenetic tree was visualized and annotated using the online phylogenetic tree visualization tool iTOL.^1^

### 2.5 Genotypic drug resistance prediction

We utilized the web-based tool TBProfiler (version 4.3.0) to analyze *M. tuberculosis* WGS data for drug resistance prediction (Phelan et al., 2019). Drug resistance was predicted using the curated drug-resistance Tuberculosis Database within TBProfiler. This database has undergone extensive testing on over 17,000 samples with genotypic and phenotypic data. The resistance-associated polymorphisms (SNPs and indels) identified by TBProfiler were further evaluated based on the WHO-endorsed catalog of molecular targets for *M. tuberculosis* complex drug-susceptibility testing and resistance interpretation (Walker et al., 2022). This additional assessment ensures reliable and accurate interpretation of drug resistance profiles. For more detailed information on the predicted drug resistance results, please refer to Supplementary Table 3.

### 2.6 Propagation analysis

To explore the influence of mutations in toxin-antitoxin system genes on the transmission of *M. tuberculosis*, we conducted analyses on transmission clusters and transmission clades (Seto et al., 2017). Building upon prior research (Walker et al., 2013), we defined genome-based transmission clusters as pairs of isolates separated by ≤12 SNPs. Genome-based transmission clades were defined as pairs of isolates separated by ≤25 SNPs. To classify the transmission clades into different categories, we adopted a classification system established by previous scholars. The transmission clades were categorized into three groups based on their size: large (above the 75th percentile), medium (between the 25th and 75th percentiles), and small (below the 25th percentile) (Chiner-Oms et al., 2019). For a comprehensive analysis of global distribution patterns and transmission dynamics among *M. tuberculosis* isolates, we classified them into two main groups: cross-country clades and within-country clades. Cross-country clades consisted of isolates originating from two or more different countries. Additionally, we further classified the *M. tuberculosis* isolates into cross-regional and within-regional clades based on their geographic location, using the United Nations standard regions (UN M.49). Cross-regional clades comprised isolates from two or more different regions.

### 2.7 Acquisition of toxin-antitoxin system genes

Initially, our analysis started with the retrieval of all genes correlated with *Mycobacterium tuberculosis* from the NCBI database, which yielded a comprehensive set of 4,015 genes. We concentrated our study on the specific strain, *Mycobacterium tuberculosis H37Rv*, and meticulously filtered that list down to 4,009 genes, guided by their respective organism names. Subsequently, our attention was directed toward refining the gene selection, with a focus on identifying those associated with the toxin-antitoxin system. This involved evaluating their functional descriptions and characteristic annotations, resulting in the successful identification of 78 genes directly implicated in the toxin-antitoxin system. To further our investigation on these genes, we employed Python, a versatile programming language with robust data analysis capabilities, to identify mutations within the set of toxin-antitoxin system genes (Supplementary Table 4).

### 2.8 Statistical analysis and modeling

Categorical data were presented as frequencies and percentages. In order to improve statistical reliability, Mutations observed fewer than 10 times were discarded prior to continuing analysis. Statistical analyses were performed by generalized linear mixed models (GLMM) in R (version 4.2.3). In addition, Python 3.7.4 with the Scikit-learn library was used to implement random forest and gradient boosting decision tree algorithms for further data analysis. To evaluate the performance of the models, all samples were randomly divided into training and test sets at a ratio of 7:3. Various metrics such as Kappa, sensitivity, specificity, accuracy, positive predictive value (PPV), negative predictive value (NPV), positive likelihood ratio (PLR), negative likelihood ratio (NLR), and area under curve (AUC) were calculated to assess the models’ effectiveness (Luo et al., 2022). Importantly, after fitting the models, we assessed the importance of input variables on the model’s predictions. By assigning scores to each input feature, we identified the top-performing variables by taking the intersection of both conditions. This approach allowed us to identify the most influential features contributing to the precision of predicting risk factors (Bi et al., 2018; Agarwal et al., 2019). All models included lineage and geographical location as covariates to correct for potential confounding factors. All statistical tests were two-tailed, with *p*-values less than 0.05 considered statistically significant.

## 3 Results

### 3.1 Characteristics of study samples

**Figure 1:**
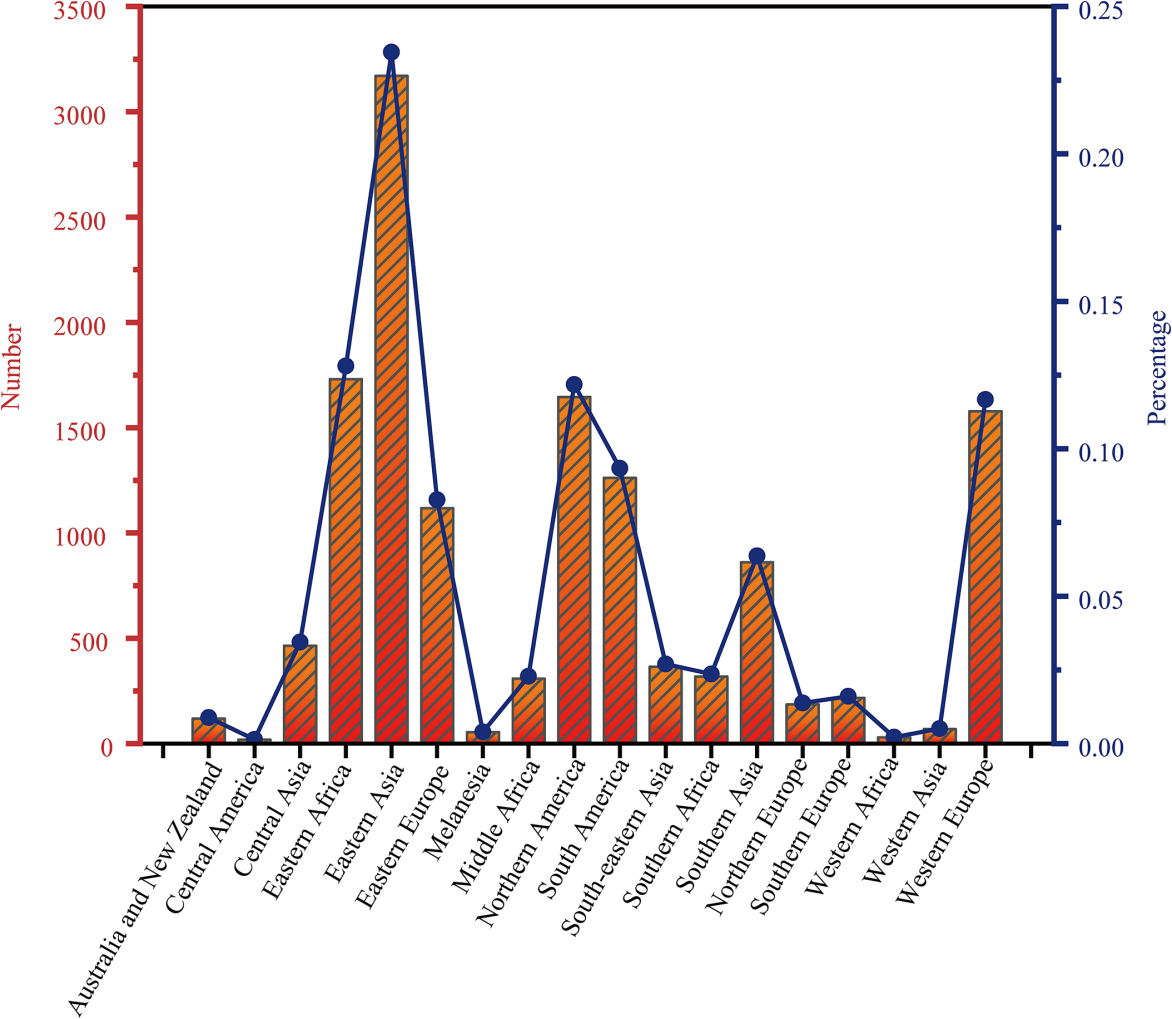
Distribution of 13,518 strains of *Mycobacterium tuberculosis* in 18 regions of the world.

**Figure 2:**
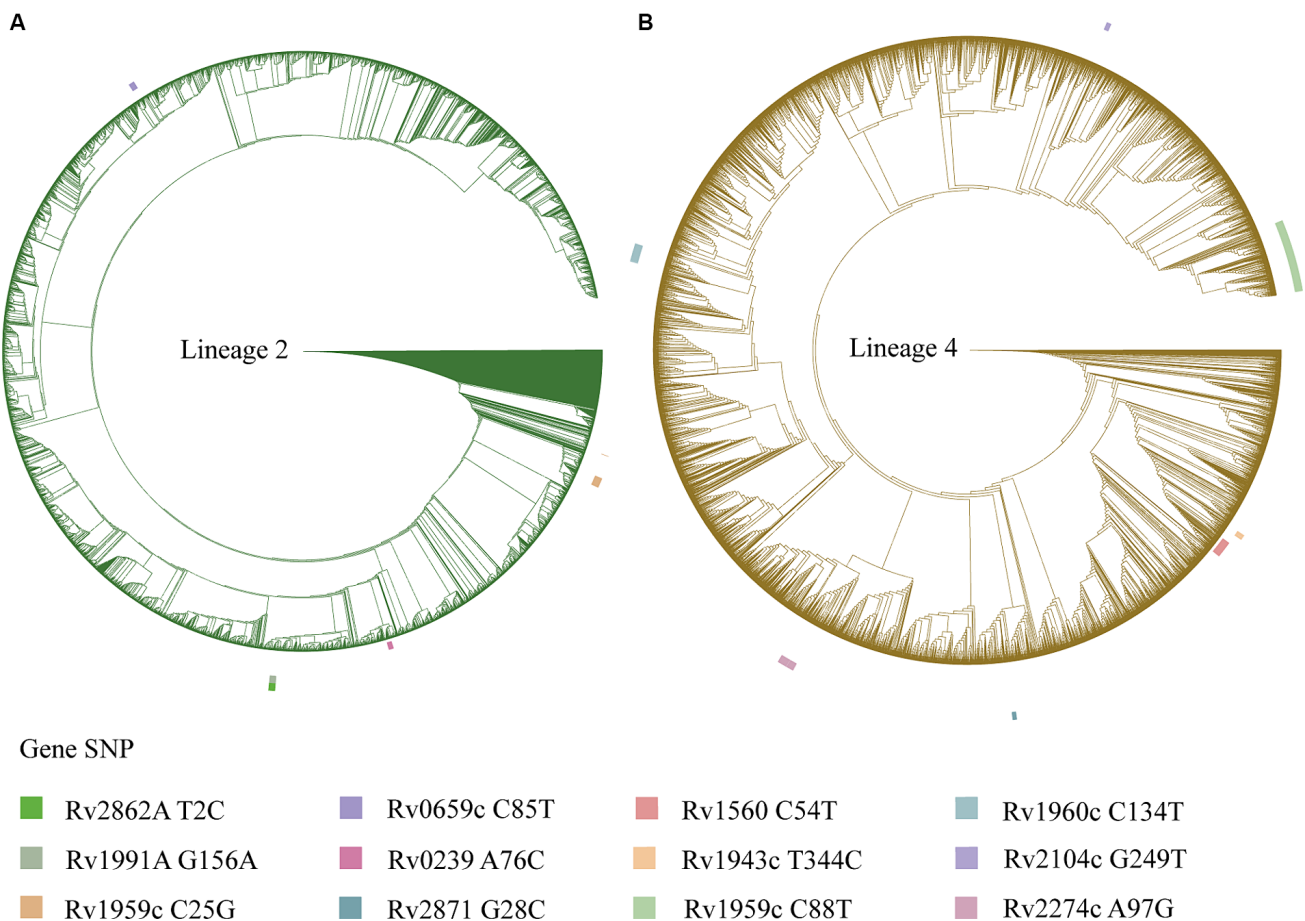
A phylogenetic tree is depicted for the *Mycobacterium tuberculosis* isolates, with the outer circle indicating mutation sites of the toxin-antitoxin system genes. **(A)** Phylogenetic tree for the *Mycobacterium tuberculosis* isolates of lineage 2. **(B)** Phylogenetic tree for the *Mycobacterium tuberculosis* isolates of lineage 4.

A total of 13,518 isolates were included in this study. We identified a total of 70,346 SNPs related to the toxin-antitoxin system. Out of the included strains, 6,488 (48%) belonged to lineage 4, 5,133 (37.97%) belonged to lineage 2, and only 10 strains (0.07%) belonged to lineage 6, while 29 strains (0.21%) belonged to lineage 7. By dividing the isolates into clusters based on 12 SNPs, a total of 5,717 strains clustered together, resulting in a clustering rate of 0.42. The *M. tuberculosis* isolates were further categorized into 1,955 clusters, with the number of isolates per cluster ranging from 2 to 146. Among the lineage 4 group, 3,245 (50.02%) isolates formed clusters, while within the lineage 2 group, 2,043 (39.80%) isolates formed clusters. Additionally, the majority of the *M. tuberculosis* strains analyzed in this study originated from Eastern Asia (*n* = 3,170, 23.45%) and Northern America (*n* = 1,646, 12.18%). Other regions contributing substantial sample sizes include Eastern Africa (*n* = 1731, 12.81%), Western Europe (*n* = 1,578, 11.67%), Northern Europe (*n* = 1,262, 9.34%), and Eastern Europe (*n* = 1,118, 8.27%), see Figure 1. Applying a threshold of 25 SNPs for clades, a total of 7,808 isolates claded together, resulting in a clading rate of 0.58. The *M. tuberculosis* isolates were further grouped into 2,218 clades, with the number of isolates per clade ranging from 2 to 192. Among these clades, there were 187 cross-country clades, consisting of 2 to 3 countries per clade, and 164 cross-regional clades, consisting of 2 to 3 regions per clade, as shown in Table 1. The phylogenetic tree of *M. tuberculosis* isolates was constructed as described in Figure 2.

**Table 1:** The characteristics of *Mycobacterium tuberculosis* isolates.

Characteristic	Number of isolates (%)	
Lineage		
Lineage 1	851 (6.30)	
Lineage 2	5,133 (37.97)	
Lineage 3	969 (7.17)	
Lineage 4	6,488 (48)	
Lineage 5	38 (0.28)	
Lineage 6	10 (0.07)	
Lineage 7	29 (0.21)	
12 SNPs		
Cluster	5,717 (42.29)	
No-cluster	7,807 (57.71)	
Lineage 2	cluster	2043 (39.80)
	no-cluster	3,090 (60.20)
Lineage 4	cluster	3,245 (50.02)
	no-cluster	3,243 (49.98)
25 SNPs		
Clade	7,808 (57.76)	
No-Clade	5,710 (42.24)	
Cross_country	Yes	704 (9.02)
	No	7,104 (90.98)
Cross_regional	Yes	650 (8.32)
	No	7,158 (91.68)
Clades by size	Small	2,548 (32.63)
	Medium	3,264 (41.80)
	Large	1996 (25.56)

### 3.2 Relationship between toxin-antitoxin system gene mutations and transmission clusters

**Figure 3:**
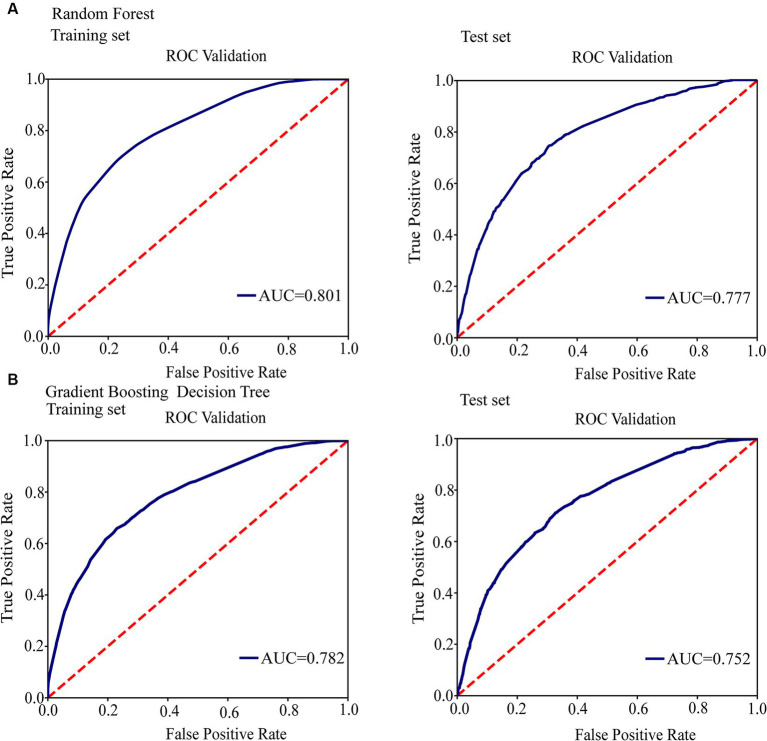
Conduct ROC curve analysis to evaluate the performance of models for the relationship between mutations in toxin-antitoxin system genes and transmission clusters. **(A)** ROC analysis showing the performance of the random forest model. **(B)** ROC analysis showing the performance of the gradient boosting decision tree.

We conducted a filtering process to exclude sites with less than 10 mutations, resulting in a final selection of 182 SNPs for further analysis. Our investigation aimed to explore the correlation between these 182 SNPs and clustering by comparing isolates within clusters to those outside clusters. The generalized linear mixed model (GLMM) revealed that 27 SNPs were statistically significant for clustering (*p* < 0.05) (Supplementary Table 5). Among these significant SNPs, there were 18 nonsynonymous SNPs, one start lost site, one stop gained site, and seven synonymous SNPs. Notably, these genetic variations showed a positive correlation with transmission clusters in *M. tuberculosis* isolates, see Table 2 for details. Furthermore, we employed random forest and gradient boosting decision tree models to establish prediction models (Table 3; Figure 3; Supplementary Table 14). However, the SNPs *Rv0298* G213A, *Rv1103c* G56A, and *Rv2871* G28C did not contribute significantly to the gradient boosting decision tree model. In summary, our findings suggested that the presence of *Rv0064A* (*vapB1*, G34A), *Rv0239* (*vapB24*, A76C),*Rv0300* (*vapB2*, T171C), *Rv0659c* (*mazF2*, C85T), *Rv0660c* (*mazE2*, G104A), *Rv0748* (*vapB31*, T112C), *Rv1247c* (*relB*, T226A), *Rv1560* (*vapB11*, C54T), *Rv1943c* (*mazE5*, T344C), *Rv1952* (*vapB1*4, A29G), *Rv1959c* (*parE1*, C103T, C88T), *Rv1960c* (*parD1*, C134T), *Rv1991A* (*mazE6*, G156A), *Rv2009* (*vapB1*5, T6C, G237A), *Rv2142c* (*parE2*, C48G), *Rv2142A* (*parD2*, A196G), *Rv2274c* (*mazF8*, A97G), *Rv2526* (*vapB1*7, G213C), *Rv2550c* (*vapB2*0, A54C), *Rv2654c* T152C, *Rv2862A* (*vapB23*, T2C), and *Rv3385c* (*vapB46*, G70A) were positively associated with transmission clusters in *M. tuberculosis* isolates.

**Table 2:** Positive correlation between toxin-antitoxin system gene mutations and transmission clusters.

Rv number	Gene	Position	SNP	Amino acid changes	Generalized linear mixed model		Random forest	Gradient boosted classification tree
					*p* value	OR (95%CI)	Importance score	Importance score
Rv0064A	vapB1	71,622	G34A	Asp12Asn	0.001	3.241 (1.596–6.581)	0.00289	0.00120
Rv0239	vapB24	289,179	A76C	Thr26Pro	0.001	18.331 (3.116–107.848)	0.00364	0.00530
Rv0298	-	363,464	G213A	Arg71Arg	0.041	4.802 (1.068–21.597)	0.00868	0
Rv0300	vapB2	363,996	T171C	Gly57Gly	0.009	3.387 (1.354–8.476)	0.00313	0.00120
Rv0659c	mazF2	754,909	C85T	Arg29Cys	0.015	3.678 (1.283–10.545)	0.00296	0.00040
Rv0660c	mazE2	755,122	G104A	Arg35His	2.82E-04	4.474 (1.993–10.045)	0.01997	0.03120
Rv0748	vapB31	841,058	T112C	Phe38Leu	0.01	19.632 (2.051–187.954)	0.00189	0.00190
Rv1103c	mazE3	1,231,236	G56A	Gly19Asp	0.031	4.242 (1.139–15.798)	0.00030	0
Rv1247c	relB	1,389,019	T226A	Phe76Ile	0.006	8.273 (1.827–37.465)	0.00072	0.00270
Rv1560	vapB11	1,764,808	C54T	Ala18Ala	4.78E-08	15.895 (5.888–42.909)	0.00348	0.00100
Rv1943c	mazE5	2,195,004	T344C	Leu115Pro	0.006	17.911 (2.282–140.553)	0.00319	0.00250
Rv1952	vapB14	2,200,754	A29G	Lys10Arg	0.001	2.262 (1.383–3.7)	0.00959	0.00360
Rv1959c	parE1	2,203,875	C103T	Leu35Leu	0.004	2.254 (1.304–3.895)	0.00730	0.00520
Rv1959c	parE1	2,203,890	C88T	Gln30*	4.27E-12	8.558 (4.662–15.709)	0.01542	0.01690
Rv1960c	parD1	2,204,092	C134T	Thr45Ile	1.08E-04	4.986 (2.211–11.244)	0.00559	0.00600
Rv1991A	mazE6	2,234,736	G156A	Arg52Arg	8.65E-05	9.605 (3.104–29.72)	0.00468	0.00500
Rv2009	vapB15	2,258,035	T6C	Tyr2Tyr	4.42E-05	4.692 (2.235–9.853)	0.00167	0.00340
Rv2009	vapB15	2,258,266	G237A	Glu79Glu	0.014	2.765 (1.231–6.207)	0.00385	0.00170
Rv2142c	parE2	2,402,463	C48G	Phe16Leu	7.44E-06	6.461 (2.857–14.612)	0.00572	0.00910
Rv2142A	parD2	2,402,527	A196G	Ile66Val	0.011	3.531 (1.337–9.327)	0.00143	0.00040
Rv2274c	mazF8	2,546,709	A97G	Ile33Val	4.77E-07	21.31 (6.478–70.103)	0.00772	0.01290
Rv2526	vapB17	2,851,303	G213C	Glu71Asp	0.033	3.93 (1.119–13.795)	0.00187	0.00090
Rv2550c	vapB20	2,870,311	A54C	Glu18Asp	0.044	3.111 (1.033–9.375)	0.00220	0.00270
Rv2654c	-	2,977,083	T152C	Val51Ala	0.049	2.795 (1.002–7.795)	0.00151	0.00030
Rv2862A	vapB23	3,174,748	T2C	Ile1?	0.005	2.271 (1.273–4.05)	0.00280	0.00130
Rv2871	vapB43	3,183,165	G28C	Glu10Gln	0.005	6.967 (1.816–26.73)	0.00277	0
Rv3385c	vapB46	3,799,874	G70A	Ala24Thr	0.002	2.74 (1.427–5.263)	0.00203	0.00140

OR, odds ratio; CI, confidence interval. *Represents a stop SNP.

**Table 3:** The performance of various models for discriminating clustered isolates from non-clustered isolates.

Parameters	Training set (*n* = 9,462, 3,998 clustered isolates, 5,464 non-clustered isolates)		Test set (*n* = 4,056, 1719 clustered isolates, 2,337 non-clustered isolates)	
	Random forest	Gradient boosted classification tree	Random forest	Gradient boosted classification tree
Kappa	0.447	0.43	0.414	0.371
AUC	0.801	0.782	0.777	0.752
(95% CI)	(0.793, 0.809)	(0.774, 0.79)	(0.764, 0.79)	(0.739, 0.765)
Sensitivity	0.625	0.614	0.602	0.586
(95% CI)	(0.615, 0.635)	(0.604, 0.624)	(0.587, 0.617)	(0.571, 0.601)
Specificity	0.815	0.809	0.804	0.779
(95% CI)	(0.807, 0.823)	(0.801, 0.817)	(0.792, 0.816)	(0.766, 0.792)
PPV	0.712	0.701	0.694	0.663
(95% CI)	(0.703, 0.721)	(0.692, 0.71)	(0.68, 0.708)	(0.648, 0.678)
NPV	0.748	0.742	0.733	0.717
(95% CI)	(0.739, 0.757)	(0.733, 0.751)	(0.719, 0.747)	(0.703, 0.731)
PLR	2.826	2.717	2.6	2.346
(95% CI)	(2.811, 2.841)	(2.702, 2.732)	(2.577, 2.623)	(2.322, 2.37)
NIR	0.354	0.368	0.385	0.426
(95% CI)	(0.321, 0.387)	(0.335, 0.401)	(0.336, 0.434)	(0.38, 0.472)
Accuracy	0.735	0.727	0.719	0.697
(95% CI)	(0.726, 0.744)	(0.718, 0.736)	(0.705, 0.733)	(0.683, 0.711)

AUC, area under the curve; PPV, positive predictive value; NPV, negative predictive value; PLR, positive likelihood ratio; NLR, negative likelihood ratio; CI, confidence.

### 3.3 Relationship between toxin-antitoxin system gene mutations and transmission clusters of lineages

After excluding sites with less than 10 mutations, a total of 46 SNPs were identified and included for further analysis. Specifically focusing on clustered isolates belonging to lineage 2, we investigated the relationship between these 46 SNPs and non-clustered isolates. The GLMM analysis revealed that five SNPs showed statistical significance for clustering (*p* < 0.05) (Supplementary Table 6). Among these significant SNPs, there were three nonsynonymous SNPs, one start lost site, and one synonymous SNP, all of which displayed a positive correlation with clustering. Notably, these significant SNPs included Rv0239 (*vapB24*, A76C), *Rv0659c* (*mazF2*, C85T), *Rv1959c* (*parE1*, C25G), *Rv1991A* (*mazE6*, G156A), and *Rv2862A* (*vapB23*, T2C). Furthermore, prediction models were established using random forest and gradient boosting decision tree algorithms (Supplementary Tables 10, 15; Supplementary Figure 1). The findings demonstrated that *vapB24* A76C, *mazF2* C85T, *parE1* C25G, *mazE6* G156A, and *vapB23* T2C significantly contributed to both the random forest and gradient boosting decision tree models. Overall, our results indicated a positive correlation between the SNPs *vapB24* A76C, *mazF2* C85T, *parE1* C25G, *mazE6* G156A, *vapB23* T2C and transmission clusters within *M. tuberculosis* isolates of lineage 2.

After filtering out sites with less than 10 mutations, we selected a total of 82 SNPs for further analysis. Our focus was specifically on clustered isolates belonging to lineage 4, and we aimed to investigate the relationship between these 82 SNPs and clustered isolates. Using the GLMM analysis, we identified 17 SNPs that showed statistical significance for clustering (*p* < 0.05) (Supplementary Table 7). Among these significant SNPs, 11 were nonsynonymous SNPs, one was a stop gained SNP, and five were synonymous SNPs, all exhibiting a positive correlation with clustering, see Table 4 for details. Furthermore, we established prediction models using random forest and gradient boosting decision tree algorithms (Supplementary Tables 11, 16; Supplementary Figure 2). However, the SNPs *Rv0064A* G34A, *Rv2009* T6C, *Rv2104c* G249T, and *Rv3385c* G70A did not contribute significantly to the gradient boosting decision tree model. In summary, our findings indicated a positive correlation between the SNPs *Rv0300* (*vapB2*, T171C), *Rv0660c* (*mazE2*, G104A), *Rv1560* (*vapB11*, C54T), *Rv1943c* (*mazE5*, T344C), *Rv1952* (*vapB1*4, A29G), *Rv1959c* (*parE1*, C88T), *Rv1960c* (*parD1*, C134T), *Rv2009* (*vapB1*5, G237A), *Rv2142c* (*parE2*, C48G), *Rv2274c* (*mazF8*, A97G), *Rv2526* (*vapB1*7, G213C), *Rv2550c* (*vapB2*0, A54C), *Rv2871* (*vapB43*, G28C), and transmission clusters within lineage 4 of *M. tuberculosis* isolates.

**Table 4:** Positive correlation between toxin-antitoxin system gene mutations and transmission clusters of lineage4.

Rv number	Gene	Position	SNP	Amino acid changes	Generalized linear mixed model		Random forest	Gradient boosted classification tree
					*p* value	OR (95%CI)	Importance score	Importance score
Rv0064A	vapB1	71,622	G34A	Asp12Asn	0.005	2.806 (1.371–5.741)	0.00503	0
Rv0300	vapB2	363,996	T171C	Gly57Gly	0.024	2.818 (1.144–6.94)	0.00791	0.00400
Rv0660c	mazE2	755,122	G104A	Arg35His	0.006	3.115 (1.378–7.038)	0.04155	0.09480
Rv1560	vapB11	1,764,808	C54T	Ala18Ala	6.78E-06	10.003 (3.669–27.271)	0.00691	0.00690
Rv1943c	mazE5	2,195,004	T344C	Leu115Pro	0.005	19.158 (2.435–150.733)	0.00847	0.00890
Rv1952	vapB14	2,200,754	A29G	Lys10Arg	0.002	2.219 (1.357–3.63)	0.02088	0.00870
Rv1959c	parE1	2,203,890	C88T	Gln30*	5.10E-13	10.267 (5.457–19.315)	0.03006	0.05750
Rv1960c	parD1	2,204,092	C134T	Thr45Ile	0.001	4.27 (1.862–9.791)	0.01080	0.00620
Rv2009	vapB15	2,258,035	T6C	Tyr2Tyr	0.04	2.218 (1.036–4.753)	0.00410	0
Rv2009	vapB15	2,258,266	G237A	Glu79Glu	0.008	2.993 (1.333–6.721)	0.00346	0.00770
Rv2104c	vapB37	2,364,533	G249T	Gly83Gly	0.006	8.238 (1.804–37.608)	0.00494	0
Rv2142c	parE2	2,402,463	C48G	Phe16Leu	0.005	3.301 (1.438–7.575)	0.00375	0.00340
Rv2274c	mazF8	2,546,709	A97G	Ile33Val	0.001	29.592 (3.984–219.821)	0.01375	0.02100
Rv2526	vapB17	2,851,303	G213C	Glu71Asp	0.037	3.828 (1.087–13.487)	0.00452	0.00050
Rv2550c	vapB20	2,870,311	A54C	Glu18Asp	0.02	3.792 (1.228–11.705)	0.00432	0.00080
Rv2871	vapB43	3,183,165	G28C	Glu10Gln	0.004	7.272 (1.882–28.103)	0.00923	0.00140
Rv3385c	vapB46	3,799,874	G70A	Ala24Thr	0.041	2.014 (1.03–3.937)	0.00303	0.00000

OR, odds ratio; CI, confidence interval. *Represents a stop SNP.

### 3.4 Relationship between toxin-antitoxin system gene mutations and cross-country transmission

After excluding sites with fewer than 10 mutations, a total of 128 SNPs within genes associated with the toxin-antitoxin system were identified and included for analysis. The objective was to investigate the relationship between these SNPs and cross-country transmission clades. The GLMM analysis revealed that seven nonsynonymous SNPs exhibited statistical significance for cross-country transmission clades (*p* < 0.05) (Supplementary Table 8). These significant SNPs included *Rv0657c* (*vapB6*, A29C), *Rv0748* (*vapB31*, T112C), *Rv1960c* (*parD1*, C134T), *Rv2104c* (*vapB37*, G205C), *Rv2547* (*vapB1*9, C188T), *Rv2653c* A80C, and *Rv2830c* (*vapB2*2, C167T). Additionally, random forest and gradient boosting decision tree models were employed to establish prediction models for these SNPs (Supplementary Tables 11, 17; Supplementary Figure 3). The results indicated that *vapB6* A29C, *vapB31* T112C, *parD1* C134T, *vapB37* G205C, *Rv2653c* A80C, and *vapB2*2 C167T made significant contributions to both the random forest and gradient boosting decision tree models. However, the SNP *vapB1*9 C188T did not contribute significantly to the gradient boosting decision tree model. Overall, our results showed that *vapB6* A29C, *vapB31* T112C, *parD1* C134T, *vapB37* G205C, *Rv2653c* A80C, and *vapB2*2 C167T were positively correlated with transmission clades across different countries.

### 3.5 Relationship between toxin-antitoxin system gene mutations and cross-regional transmission

After excluding sites with less than 10 mutations, we identified and included a total of 128 SNPs of toxin-antitoxin system genes. The GLMM showed that seven nonsynonymous SNPs were found to be statistically significant for transmission clades of cross-country (*p* < 0.05) (Supplementary Table 9), including *Rv0657c* (*vapB6*, A29C), *Rv0748* (*vapB31*, T112C), *Rv1960c* (*parD1*, C134T), *Rv2104c* (*vapB37*, G205C), *Rv2547* (*vapB1*9, C188T), *Rv2653c* A80C, *Rv2830c* (*vapB2*2, C167T). Two prediction models were established using random forest and gradient boosting decision tree (Supplementary Tables 13, 18; Supplementary Figure 4), we found that *vapB6* A29C, *vapB31* T112C, *parD1* C134T, *vapB37* G205C, *vapB1*9 C188T and *Rv2653c* A80C also contributed most to the random forest and gradient boosting decision tree. However, the SNP of *vapB2*2 C167T did not contribute significantly to the gradient boosting decision tree model. Overall, our results showed that *vapB6* A29C, *vapB31* T112C, *parD1* C134T, *vapB37* G205C, *vapB1*9 C188T, and *Rv2653c* A80C were positively correlated with transmission clades across different regions.

### 3.6 Relationship between toxin-antitoxin system gene mutations and clade size

After excluding sites with less than 10 mutations, a total of 128 SNPs within the toxin-antitoxin system were identified and included for analysis. The results revealed that 32 SNPs were significantly associated with clade size (*p* < 0.05). Among these significant SNPs, there were 21 nonsynonymous SNPs, two stop gained SNPs, and nine synonymous SNPs, all of which displayed a positive correlation with clade size. Notable examples include *vapB1* G34A, *mazE2* G104A, *vapB11* C54T, *mazE5* T344C, *vapB14* A29G, *parE1* C88T, *parD1* C134T, *vapB1*5 T6C, *parE2* C48G, *mazF8* A97G, and *vapB46* G70A. For more detailed information, please refer to Supplementary Figure 5.

## 4 Discussion

Consistent with prior research findings, our study further emphasizes the diverse functionality of TA systems in *M. tuberculosis*. These redundant TA systems serve as a backup mechanism enabling cellular adaptation and survival under adverse conditions (Min et al., 2012). They play a critical role in *M. tuberculosis*’s stress response, including nutrient deprivation, by regulating essential cellular processes like DNA replication, protein translation, and cell division. Moreover, TA systems contribute to the formation of drug resistance and persistence in *M. tuberculosis*. However, it is important to acknowledge that certain studies have reported conflicting results regarding the specific contributions of TA systems to persistence formation and stress conditions (Yu et al., 2020; Sharma et al., 2021). These discrepancies may arise from variations in experimental setups or genetic differences among *M. tuberculosis* strains used in different investigations. Therefore, additional research is needed to precisely determine the roles of TA systems in persistence formation, stress responses, and their impact on *M. tuberculosis* pathogenesis. In our study, we focused on examining the relationship between gene mutations in toxin-antitoxin systems and the transmission dynamics of *M. tuberculosis*. The *M. tuberculosis* genome contains numerous toxin-antitoxin systems, including *VapBC*, *MazEF*, *ParDE*, and *RelBE* (Ramage et al., 2009; Tandon et al., 2019). To gain deeper insights into the significance of these toxin-antitoxin systems in *M. tuberculosis* transmission, we analyzed the prevalence and genetic variation of specific toxin-antitoxin system genes across various clusters and evolutionary branches. Our analysis detected multiple mutations in these genes, suggesting they could be involved in *M. tuberculosis* transmission.

In our study, we have found a strong association between SNPs in the *VapB* antitoxin-related genes and the transmission of *M. tuberculosis*. Specifically, we identified several significant SNPs that were linked to transmission, including *vapB1* G34A, *vapB24* A76C, *vapB31* T112C, *vapB14* A29G, and *vapB1*5 (T6C, G237A). We observed that *vapB24* A76C and *vapB23* T2C were particularly associated with transmission, especially in lineage 2. Additionally, *vapB2* T171C, *vapB11* C54T, *vapB14* A29G, *vapB1*5 G237A, *vapB1*7 G213C, and *vapB2*0 A54C were significantly related to transmission, especially in lineage 4. Furthermore, we found that *vapB43* G28C was associated with transmission in lineage 4, while *vapB6* A29C, *vapB31* T112C, and *vapB37* G205C were correlated with cross-country and cross-regional transmission. We also found that *vapB1* G34A, *vapB11* C54T, *vapB14* A29G, *vapB15* T6C, and *vapB46* G70A were related to clade size. The *VapBC* system is crucial for regulating the behavior and adaptation of *M. tuberculosis* under diverse environmental stresses. It comprises stable *VapC* toxins and labile *VapB* antitoxins, whose interplay is essential for bacterial growth, survival, and response to stress conditions (Robson et al., 2009; Winther and Gerdes, 2011). During periods of stress, antitoxin molecules are degraded, leading to the release of toxins, such as *VapC*, through their RNase activity (Min et al., 2012). Consequently, these toxins inhibit or slow down cellular metabolism, providing a survival advantage to the bacterium during adverse conditions. The delicate balance between *VapB* antitoxins and *VapC* toxins is crucial for maintaining bacterial homeostasis and ensuring appropriate responses to external stimuli (McKenzie et al., 2012). Overall, our study provides compelling evidence for the significant association between SNPs in *VapB* antitoxin-related genes and *M. tuberculosis* transmission. These findings shed light on the intricate role of the *VapBC* toxin-antitoxin system in regulating bacterial behavior and underscore the importance of genetic variations within this system in shaping transmission dynamics.

Our study has revealed the association between SNPs in other TA system genes and the transmission of *M. tuberculosis*. Specifically, we focused on the *MazEF* family, which consists of nine TA systems encoded in an operon (Ahmed et al., 2022). We found a close connection between the *mazE6* G156A and *mazF2* C85T gene polymorphisms and the transmission clusters, particularly within lineage 2. These variants exhibited significant correlations with the formation and expansion of transmission clusters. However, *mazE6* G156A is a synonymous mutation (Arg52Arg), which does not directly alter the protein’s function but may still affect the protein through other mechanisms. For example, in certain situations, synonymous mutations can lead to changes in transcription regulatory elements, thereby influencing gene expression levels. However, further research is needed to confirm these effects. Similarly, the *mazF2* C85T variant may alter the stability of the *MazF* and modulate the delicate balance between toxin and antitoxin interactions (Leplae et al., 2011). Furthermore, our study identified a strong correlation between the *mazE2* G104A, *mazE5* T344C, and *mazF8* A97G gene polymorphisms and the transmission clusters, especially within lineage 4 and clade size. While it’s plausible that these genetic variations influence the *MazEF* system activity, stability, and domain structure, our ability to fully elucidate these mechanisms is currently limited. Therefore, it’s crucial to interpret these functional implications cautiously and consider other potential contributory factors to *M. tuberculosis* transmission. Furthermore, no SNPs in the *MazEF* system were found to be associated with cross-country and cross-regional transmission of *M. tuberculosis* in our study. Future investigations should aim to provide a more comprehensive understanding of these effects, confirm these hypotheses, and uncover the precise impact of these mutations on the dynamics of *M. tuberculosis* transmission.

The *ParDE* toxin-antitoxin system in *M. tuberculosis* plays a crucial role in bacterial transmission dynamics. Our research has identified specific genetic variations in the parE and *parD* genes, such as *parE1* C88T, *parE2* C48G, *parE1* C103T, *parD2* A196G, *parE1* C25G, and *parD1* C134T, that are closely linked to transmission clusters, particularly within lineage 4 and lineage 2. These genetic variants impact cross-country and cross-regional transmissions, highlighting the significance of the *ParD*E system in the spread of *M. tuberculosis*. Variations in the *parD* gene, including those involving *Rv2142A* (*parD2*) and *Rv1960c* (*parD1*), can modify the activity and regulatory mechanisms of the *ParD* antitoxin. Similarly, variations in the *parE* gene, particularly those affecting *Rv1959c* (*parE1*), influence the function and stability of the *ParE* toxin, thus impacting its interaction with the *ParE* antitoxin (Xu et al., 2018). Understanding these genetic interactions is crucial for deciphering *M. tuberculosis* transmission dynamics and developing targeted interventions to effectively combat tuberculosis. Additionally, our research has identified a unique SNP, T226A, in the *relB* gene that is associated with transmission clusters in *M. tuberculosis*. This genetic variation further adds to the complexity of bacterial transmission dynamics, highlighting the intricate interplay between genetic factors and the spread of *M. tuberculosis*.

In terms of drug development and therapeutic interventions, our research findings could potentially have significant implications. The diverse functions of TA systems suggest potential targets for novel therapeutic strategies in *M. tuberculosis*. Understanding the relationship between genetic variations and functional consequences within these TA systems might help us discover new methods to disrupt or modulate their activity, thereby affecting the survival and transmission dynamics of the bacterium. Firstly, interventions targeting specific SNPs in TA systems such as *VapBC*, *MazEF*, *ParD*E, and *RelB*E could possibly directly alter the stability and activity of their toxins or antitoxins, thus impacting the growth, survival, and adaptability of *M. tuberculosis* (Robson et al., 2009; Leplae et al., 2011; Winther and Gerdes, 2011; McKenzie et al., 2012; Ahmed et al., 2022). The SNPs we discovered, including *vapB24* A76C and *vapB23* T2C, have the potential to serve as genetic markers for targeted drug design, allowing for more personalized treatment approaches. Additionally, mutations like *parE1* C88T, *parE2* C48G, and *parE1* C103T show associations with cross-national and cross-regional transmissions of *M. tuberculosis*, which could aid in the development of more effective treatment plans to reduce global transmission. However, it’s important to note that while these genetic insights hold potential, they still require experimental validation to confirm their clinical significance and functional implications. Each mutation may lead to different functional impacts, and there might be other complexities involved, such as drug tolerance or adaptability of the bacterium under different environmental conditions. Therefore, further research is needed to delve deeper into the functional impacts of these genetic variations and precisely determine their roles in new drug development and treatment strategies. It is crucial to validate these findings through rigorous experimental studies and clinical trials before implementing them in clinical practice. Future research should aim to elucidate the specific mechanisms underlying these genetic variations and their contributions to drug response and transmission dynamics. By gaining a better understanding of the functional implications, we can more accurately tailor treatment strategies and contribute to the development of more targeted and effective interventions.

Our findings emphasize that both synonymous and non-synonymous mutations can influence the transmission of *M. tuberculosis*, suggesting that synonymous mutations in TA system genes are not universally neutral, in line with prior research by Shen et al. (2022). We believe that synonymous mutations may affect mRNA stability, splicing, or secondary structure formation. Changes in these regulatory elements can influence gene expression patterns and protein folding, thereby impacting bacterial adaptability and transmission capacity. Additionally, synonymous mutations may be part of a compensatory mechanism. While synonymous mutations themselves may not directly provide selective advantages, they may be associated with compensatory changes in other regions of the genome. These compensatory mutations could restore proper interactions between proteins, maintain enzyme activity, or optimize cellular functions affected by primary mutations, ultimately enhancing transmission capacity. Although the specific mechanisms and advantages of synonymous mutations in tuberculosis transmission are not yet fully understood, we cannot overlook their potential significance. Future research should consider the functional consequences of synonymous mutations and explore their interactions with other genetic factors, including non-synonymous mutations, drug resistance mutations, or virulence determinants. In our study, we combined local and global datasets to increase sample size for robust analysis of *M. tuberculosis* genetic variations. This approach helped identify shared and distinct variants across regions, enhancing our understanding of global pathogen diversity. Despite potential limitations such as variability from different protocols and sequencing technologies, stringent quality control measures, including SNP filtering within repetitive regions, were applied to minimize biases. Our novel findings contribute valuable insights into global *M. tuberculosis* genetic characteristics, advancing knowledge on tuberculosis pathogenesis and evolution. In future research, separate and comparative analyses of local and global data can be considered to highlight region-specific variations.

In our study, we investigated the impact of mutations in TA system genes on tuberculosis transmission. However, it is crucial to acknowledge that these correlations alone do not establish a causal relationship and should be interpreted with caution. Our modeling approach has limitations, notably in addressing potential confounding factors, such as population mobility, social networks, and inter-regional interactions. These elements may influence *M. tuberculosis* transmission but were not fully integrated into our models. We recognize that our primary focus on mutations within TA system genes may have led us to overlook other significant genetic influences, including SNPs related to drug resistance mutations or virulence determinants. While our findings contribute to the growing body of knowledge regarding the impact of toxin-antitoxin system gene mutations on tuberculosis transmission, further research is necessary to explore these intersections and understand their functional significance in detail. Limitations also arise from the sheer number of genes and computational resources required, which restricted our ability to analyze SNPs beyond the scope of our current investigation. Moreover, we lack a clear understanding of the cross-interactions and mutual regulation among the TA systems of *M. tuberculosis*, adding another layer of complexity to our study. Additionally, uncertainties inherent in the phylogenetic inference method used, such as homoplasy or recombination events, can present challenges when accurately determining evolutionary relationships. Therefore, future research should consider alternative methods to validate these findings and develop a more nuanced understanding of tuberculosis transmission. Further experimental validation is necessary to confirm the specific impact of TA system gene mutations. Future investigations should focus on refining our models to account for potential biases or shortcomings, and expanding research scope to explore the functional significance of these mutations and their direct influence on tuberculosis transmission.

We also discuss the limitations of using H37Rv as a single reference genome for analyzing *M. tuberculosis* WGS data, particularly regarding virulence and transmission. Recent studies suggest that relying solely on H37Rv may not fully capture the virulence characteristics of *M. tuberculosis*. H37Rv, commonly used as a reference genome in molecular epidemiology and drug resistance studies, does not represent the genetic diversity and variations present across all *M. tuberculosis* strains. Polymorphic loci involving genes associated with pathogenicity and host immune response, such as phospholipase C, membrane lipoproteins, adenylate cyclase gene family members, and PE/PPE gene family members, show significant differences between H37Rv and clinical isolates. Several gene families, including PE/PPE, exhibit higher substitution frequencies compared to the entire genome. Widespread genetic variability is observed at these polymorphic loci among *M. tuberculosis* clinical isolates (Fleischmann et al., 2002; O’Toole and Gautam, 2017). Phylogenetic and epidemiological analyses reveal independent occurrences of these polymorphisms, suggesting selective pressures driving these changes. Future research should incorporate genome sequences of additional reference strains, especially those directly obtained from clinical isolates, to comprehensively understand factors related to *M. tuberculosis* virulence and enable further investigations. For drug resistance inference, our analysis primarily utilized the TBProfiler platform. While incorporating additional tools/methods such as PhyResSE or bioinformatic SNP analysis could enhance robustness, resource constraints limited their implementation in this study. Thus, our results should be interpreted within the context of utilizing TBProfiler alongside the WHO-endorsed catalog. Future studies with expanded resources could consider alternative tools/methods for validation and complementation.

## 5 Conclusion

The results of this study suggest that mutations in toxin-antitoxin genes may increase the risk of *M. tuberculosis* transmission, underscoring the significance of conducting further research to explore the impact of these mutations on *M. tuberculosis* control and transmission. These findings offer new insights into the development of drug treatment strategies against tuberculosis.

## Data availability statement

The whole genome sequences have been submitted to the NCBI under the accession number PRJNA1002108.

## Author contributions

YH: Conceptualization, Formal analysis, Software, Writing – original draft, Writing – review & editing. YiL: Conceptualization, Formal analysis, Methodology, Writing – review & editing. NT: Conceptualization, Formal analysis, Investigation, Project administration, Software, Validation, Writing – review & editing. XK: Conceptualization, Data curation, Formal analysis, Investigation, Methodology, Software, Validation, Writing – original draft. YamL: Conceptualization, Data curation, Formal analysis, Investigation, Methodology, Project administration, Software, Supervision, Validation, Writing – review & editing. YaoL: Data curation, Formal analysis, Methodology, Project administration, Supervision, Validation, Writing – review & editing. HL: Conceptualization, Data curation, Funding acquisition, Investigation, Resources, Visualization, Writing – original draft, Writing – review & editing. ZW: Conceptualization, Data curation, Formal analysis, Funding acquisition, Investigation, Project administration, Resources, Software, Supervision, Visualization, Writing – original draft.

## Footnotes

^1^ https://itol.embl.de/
