# Supplementary material for: Toxin-antitoxin system gene mutations driving Mycobacterium tuberculosis transmission revealed by whole genome sequencing
Source: Front Microbiol. 2024 Jul 31;15:1398886. doi: 10.3389/fmicb.2024.1398886 (PMC11322068; doi:10.3389/fmicb.2024.1398886)
Supplement: Supplementary file 1 [file Data_Sheet_1.docx]

Supplementary Material

Toxin-Antitoxin system gene mutations driving *Mycobacterium tuberculosis* transmission revealed by Whole genome sequencing

**Yawei Hou1, Yifan Li2, Ningning Tao3, Xianglong Kong4, Yameng Li5, Yao Liu3*, Huaichen Li 3*and Zhenguo Wang1***

*** Correspondence:** Zhenguo Wang zhenguow@126.com

## Supplementary Figures


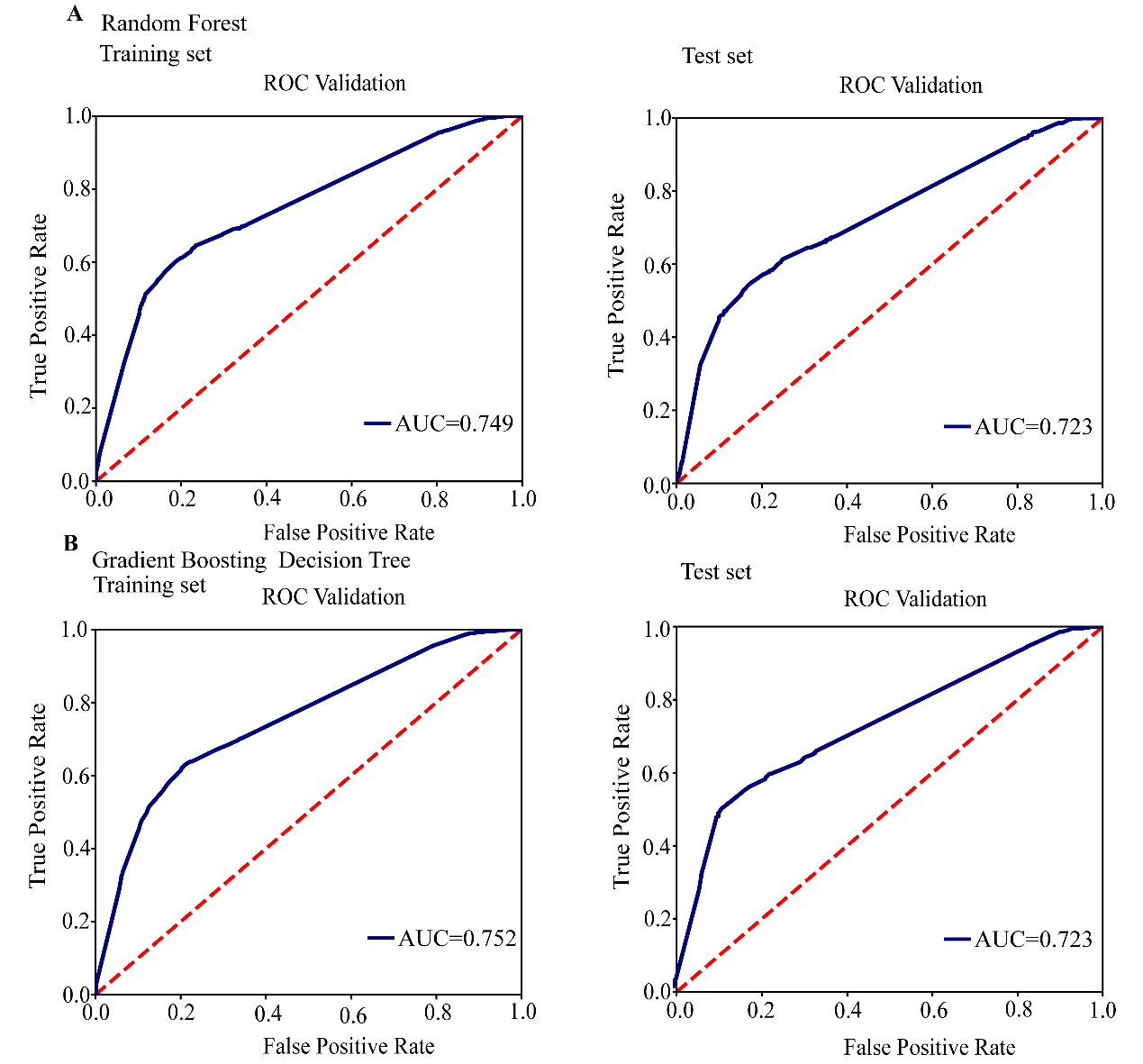


**Supplementary Figure 1.** ROC curve analysis was conducted to evaluate the performance of models for cluster analysis within lineage 2. (A) ROC analysis showing the performance of the random forest model. (B) ROC analysis showing the performance of the gradient boosting decision tree.


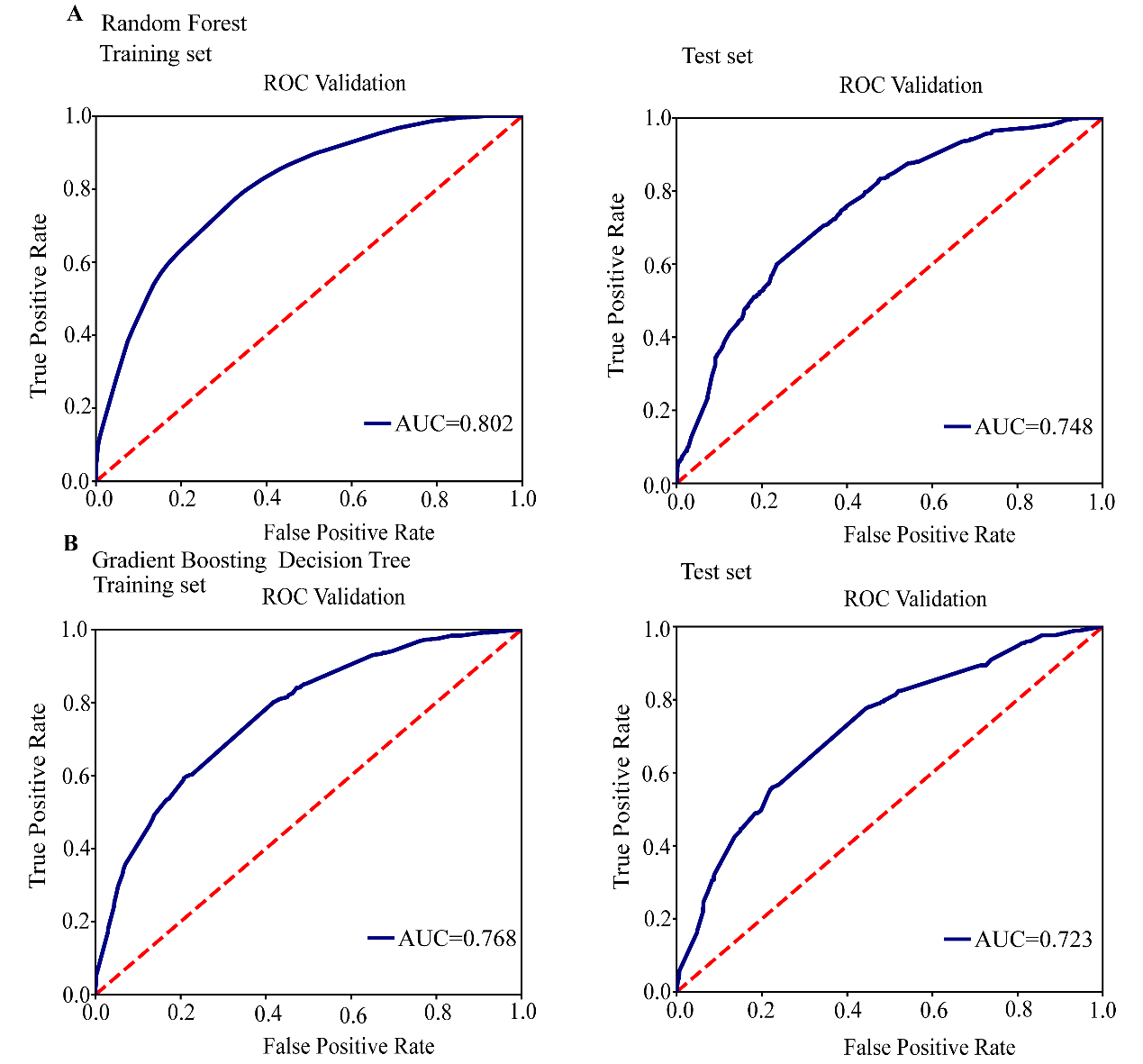


**Supplementary Figure 2.** ROC curve analysis was conducted to evaluate the performance of models for cluster analysis within lineage 4. (A) ROC analysis showing the performance of the random forest model. (B) ROC analysis showing the performance of the gradient boosting decision tree.


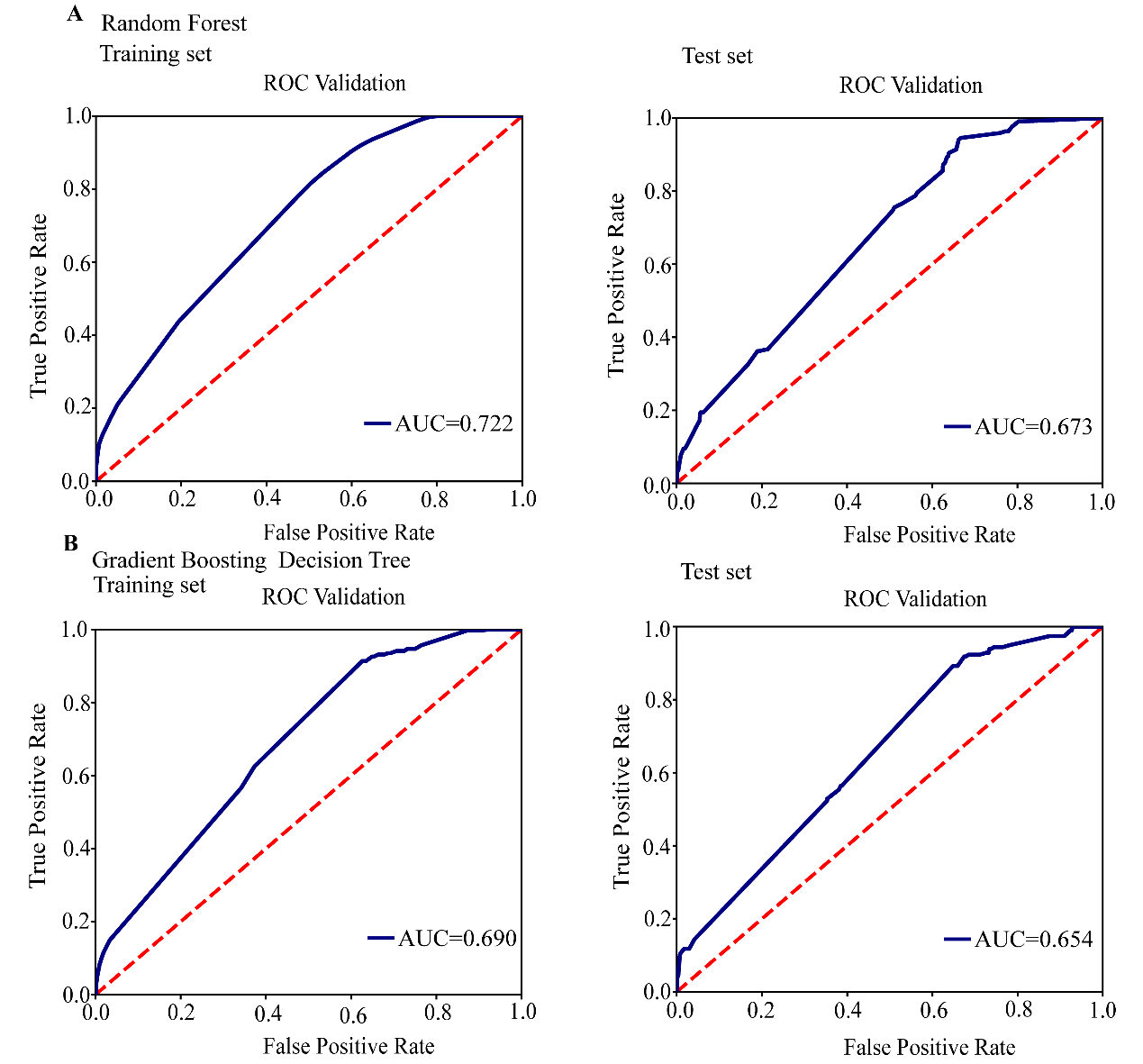


**Supplementary Figure 3.** The ROC curve analysis was conducted to evaluate the performance of models for cross-country transmission analysis. (A) ROC analysis showing the performance of the random forest model. (B) ROC analysis showing the performance of the gradient boosting decision tree.


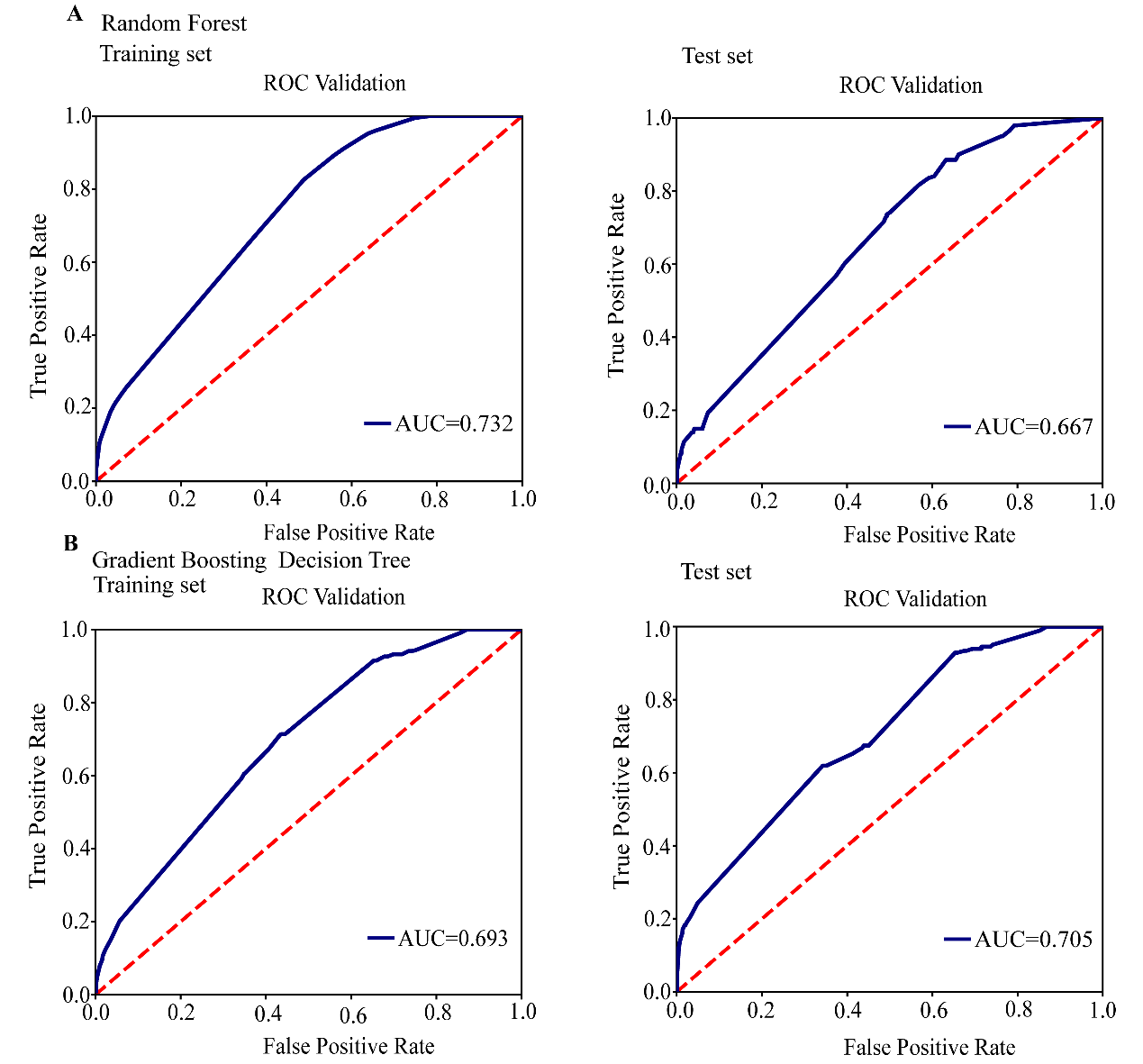


**Supplementary Figure 4.** The ROC curve analysis was conducted to evaluate the performance of models for cross-regional transmission analysis. (A) ROC analysis showing the performance of the random forest model. (B) ROC analysis showing the performance of the gradient boosting decision tree.


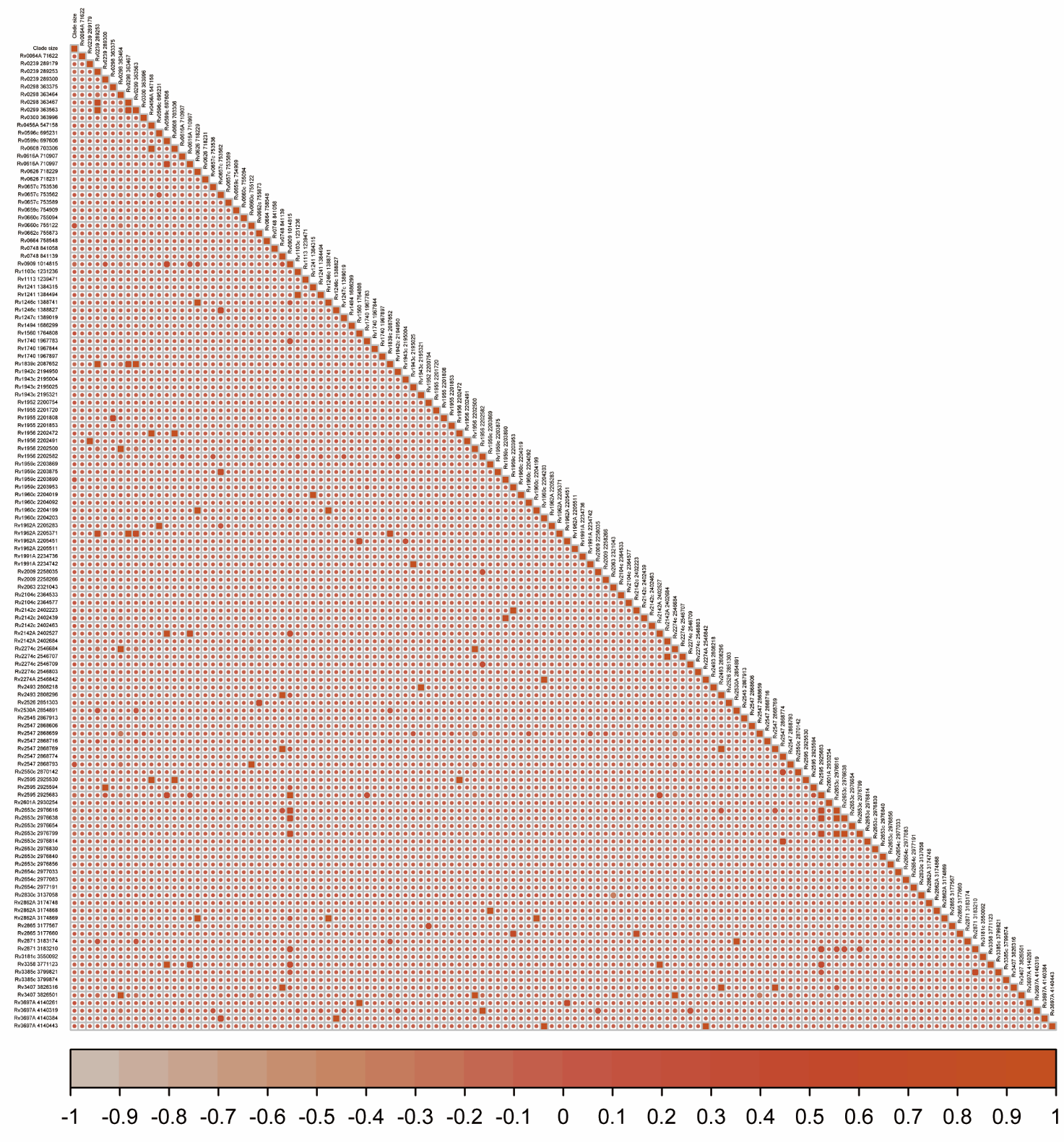


**Supplementary Figure 5.** Correlation analysis of toxin-antitoxin system gene mutations and clade size. Larger and darker points indicate stronger correlations, while smaller and lighter points represent weaker correlations. Positive correlations are shown in shades of dark red, while negative correlations are depicted in shades of light red.
