# Supplementary material for: Toxin-antitoxin system gene mutations driving Mycobacterium tuberculosis transmission revealed by whole genome sequencing
Source: Front Microbiol. 2024 Jul 31;15:1398886. doi: 10.3389/fmicb.2024.1398886 (PMC11322068; doi:10.3389/fmicb.2024.1398886)
Supplement: Supplementary file 6 [file Data_Sheet_6.docx]

Supplementary Material

Toxin-Antitoxin system gene mutations driving *Mycobacterium tuberculosis* transmission revealed by Whole genome sequencing

**Yawei Hou1, Yifan Li2, Ningning Tao3, Xianglong Kong4, Yameng Li5, Yao Liu3*, Huaichen Li 3*and Zhenguo Wang1***

*** Correspondence:** Zhenguo Wang zhenguow@126.com

# Supplementary Tables

**Supplementary Table 5** Generalized linear mixed model analysis on clustered and non-clustered isolates.

| **Rv number** | **Gene** | **Position** | **SNP** | **Amino acid changes** | **P value** | **OR(95%CI)** |
| --- | --- | --- | --- | --- | --- | --- |
| Rv0064A | vapB1 | 71622 | G34A | Asp12Asn | 0.001 | 3.241(1.596-6.581) |
| Rv0239 | vapB24 | 289179 | A76C | Thr26Pro | 0.001 | 18.331(3.116-107.848) |
| Rv0239 | vapB24 | 289253 | C150T | Asp50Asp | 0.388 | 3.382(0.212-53.842) |
| Rv0239 | vapB24 | 289300 | C197T | Ser66Phe | 0.996 | - |
| Rv0298 | - | 363375 | C124A | Leu42Met | 0.719 | 1.188(0.464-3.041) |
| Rv0298 | - | 363450 | T199G | Tyr67Asp | 0.991 | - |
| Rv0298 | - | 363464 | G213A | Arg71Arg | 0.041 | 4.802(1.068-21.597) |
| Rv0298 | - | 363467 | G216A | Ala72Ala | 0.997 | - |
| Rv0299 | - | 363563 | G88A | Ala30Thr | 1.000 | - |
| Rv0300 | vapB2 | 363861 | C36T | Asp12Asp | 0.726 | 0.748(0.147-3.795) |
| Rv0300 | vapB2 | 363883 | A58G | Ile20Val | 0.991 | - |
| Rv0300 | vapB2 | 363996 | T171C | Gly57Gly | 0.009 | 3.387(1.354-8.476) |
| Rv0456A | mazF1 | 547158 | A200G | Glu67Gly | 0.998 | - |
| Rv0550c | vapB3 | 640862 | G43A | Val15Met | 0.059 | 0.216(0.044-1.057) |
| Rv0596c | vapB4 | 695231 | G255A | Trp85* | 0.458 | 0.575(0.133-2.48) |
| Rv0599c | vapB27 | 697606 | C195T | Asp65Asp | 0.833 | 1.192(0.233-6.103) |
| Rv0608 | vapB28 | 703306 | C63T | Thr21Thr | 0.996 | - |
| Rv0608 | vapB28 | 703440 | G197A | Arg66His | 0.080 | 0.215(0.038-1.203) |
| Rv0616A | vapB29 | 710906 | G125A | Arg42His | 0.997 | - |
| Rv0616A | vapB29 | 710907 | C126G | Arg42Arg | 0.992 | - |
| Rv0616A | vapB29 | 710997 | G216A | Glu72Glu | 0.164 | 0.528(0.215-1.299) |
| Rv0626 | vapB5 | 718144 | G120A | Pro40Pro | 0.988 | 1.015(0.141-7.292) |
| Rv0626 | vapB5 | 718231 | C207T | Asn69Asn | 0.667 | 1.178(0.558-2.486) |
| Rv0657c | vapB6 | 753526 | T92G | Leu31Arg | 0.075 | 0.241(0.05-1.157) |
| Rv0657c | vapB6 | 753536 | G82A | Ala28Thr | 0.071 | 2.543(0.925-6.993) |
| Rv0657c | vapB6 | 753562 | T56C | Ile19Thr | 0.804 | 0.95(0.636-1.42) |
| Rv0657c | vapB6 | 753589 | A29C | Asp10Ala | 0.861 | 1.096(0.394-3.047) |
| Rv0659c | mazF2 | 754909 | C85T | Arg29Cys | 0.015 | 3.678(1.283-10.545) |
| Rv0659c | mazF2 | 754974 | G20A | Trp7* | 0.991 | - |
| Rv0660c | mazE2 | 755069 | C157G | Pro53Ala | 0.610 | 0.723(0.208-2.513) |
| Rv0660c | mazE2 | 755094 | G132C | Gln44His | 0.601 | 0.757(0.267-2.148) |
| Rv0660c | mazE2 | 755122 | G104A | Arg35His | 2.82E-04 | 4.474(1.993-10.045) |
| Rv0662c | vapB7 | 755873 | C151T | Arg51Cys | 0.224 | 0.661(0.339-1.289) |
| Rv0662c | vapB7 | 755947 | G77A | Arg26His | 0.441 | 0.646(0.213-1.963) |
| Rv0664 | vapB8 | 758548 | G17T | Cys6Phe | 0.634 | 1.262(0.484-3.289) |
| Rv0748 | vapB31 | 840982 | C36T | Leu12Leu | 0.724 | 1.33(0.273-6.474) |
| Rv0748 | vapB31 | 841016 | C70T | Gln24* | 0.746 | 0.787(0.185-3.351) |
| Rv0748 | vapB31 | 841039 | G93A | Glu31Glu | 0.027 | 0.053(0.004-0.711) |
| Rv0748 | vapB31 | 841058 | T112C | Phe38Leu | 0.010 | 19.632(2.051-187.954) |
| Rv0748 | vapB31 | 841180 | C234T | Gly78Gly | 0.984 | - |
| Rv0909 | - | 1014815 | T135G | His45Gln | 0.998 | - |
| Rv0910 | - | 1014893 | G28A | Val10Ile | 0.994 | - |
| Rv1103c | mazE3 | 1231236 | G56A | Gly19Asp | 0.031 | 4.242(1.139-15.798) |
| Rv1113 | vapB32 | 1239471 | C56T | Thr19Ile | 0.016 | 0.507(0.292-0.879) |
| Rv1241 | vapB33 | 1384315 | G38A | Arg13Gln | 0.815 | 1.299(0.145-11.675) |
| Rv1241 | vapB33 | 1384400 | G123T | Pro41Pro | 0.995 | - |
| Rv1246c | relE | 1388827 | A152T | Asn51Ile | 0.146 | 0.189(0.02-1.79) |
| Rv1247c | relB | 1389019 | T226A | Phe76Ile | 0.006 | 8.273(1.827-37.465) |
| Rv1398c | vapB10 | 1574765 | G3A | Met1? | 0.412 | 0.594(0.172-2.057) |
| Rv1494 | mazE4 | 1686299 | T29G | Ile10Ser | 0.007 | 0.324(0.144-0.731) |
| Rv1494 | mazE4 | 1686352 | C82T | Arg28Cys | 0.530 | 0.714(0.25-2.043) |
| Rv1494 | mazE4 | 1686408 | C138T | Ile46Ile | 0.732 | 0.773(0.177-3.378) |
| Rv1494 | mazE4 | 1686414 | C144T | Asp48Asp | 1.000 | - |
| Rv1494 | mazE4 | 1686516 | C246T | Ala82Ala | 0.730 | 1.361(0.237-7.799) |
| Rv1560 | vapB11 | 1764808 | C54T | Ala18Ala | 4.78E-08 | 15.895(5.888-42.909) |
| Rv1740 | vapB34 | 1967783 | A79G | Thr27Ala | 0.064 | 5.46(0.904-32.979) |
| Rv1740 | vapB34 | 1967844 | C140A | Ala47Glu | 0.024 | 0.612(0.399-0.938) |
| Rv1740 | vapB34 | 1967897 | G193C | Glu65Gln | 0.810 | 1.129(0.419-3.046) |
| Rv1839c | vapB13 | 2087652 | G1A | Val1? | 0.995 | - |
| Rv1839c | vapB13 | 2087847 | C66T | Arg22Arg | 0.136 | 3.15(0.697-14.23) |
| Rv1942c | mazF5 | 2194950 | A24C | Gly8Gly | 0.844 | 1.078(0.511-2.273) |
| Rv1943c | mazE5 | 2195004 | T344C | Leu115Pro | 0.006 | 17.911(2.282-140.553) |
| Rv1943c | mazE5 | 2195025 | A323G | Glu108Gly | 0.998 | - |
| Rv1943c | mazE5 | 2195321 | G27A | Thr9Thr | 0.997 | - |
| Rv1952 | vapB14 | 2200754 | A29G | Lys10Arg | 0.001 | 2.262(1.383-3.7) |
| Rv1955 | higB | 2201720 | T2C | Val1? | 0.667 | 0.876(0.479-1.603) |
| Rv1955 | higB | 2201808 | C90G | Asp30Glu | 0.799 | 0.928(0.522-1.649) |
| Rv1955 | higB | 2201853 | G135A | Gln45Gln | 0.099 | 2.036(0.874-4.741) |
| Rv1955 | higB | 2201975 | A257G | His86Arg | 0.838 | 1.153(0.294-4.524) |
| Rv1956 | higA | 2202472 | C335T | Ser112Phe | 0.996 | - |
| Rv1956 | higA | 2202491 | A354G | Ala118Ala | 0.395 | 0.561(0.148-2.126) |
| Rv1956 | higA | 2202500 | C363T | His121His | 0.052 | 7.784(0.986-61.479) |
| Rv1956 | higA | 2202582 | G445T | Ala149Ser | 0.922 | 0.883(0.074-10.594) |
| Rv1959c | parE1 | 2203704 | A274C | Met92Leu | 0.993 | - |
| Rv1959c | parE1 | 2203869 | G109A | Glu37Lys | 0.110 | 5.499(0.68-44.496) |
| Rv1959c | parE1 | 2203875 | C103T | Leu35Leu | 0.004 | 2.254(1.304-3.895) |
| Rv1959c | parE1 | 2203890 | C88T | Gln30* | 4.27E-12 | 8.558(4.662-15.709) |
| Rv1959c | parE1 | 2203953 | C25G | Pro9Ala | 0.997 | - |
| Rv1960c | parD1 | 2204019 | C207T | Asp69Asp | 0.560 | 1.813(0.245-13.422) |
| Rv1960c | parD1 | 2204092 | C134T | Thr45Ile | 1.08E-04 | 4.986(2.211-11.244) |
| Rv1960c | parD1 | 2204203 | T23C | Val8Ala | 1.000 | - |
| Rv1962A | vapB35 | 2205283 | G267A | Arg89Arg | 0.789 | 0.847(0.25-2.861) |
| Rv1962A | vapB35 | 2205371 | T179G | Val60Gly | 0.997 | - |
| Rv1962A | vapB35 | 2205451 | G99A | Lys33Lys | 0.061 | 0.712(0.5-1.016) |
| Rv1962A | vapB35 | 2205511 | G39A | Thr13Thr | 0.013 | 0.623(0.429-0.906) |
| Rv1991A | mazE6 | 2234736 | G156A | Arg52Arg | 8.65E-05 | 9.605(3.104-29.72) |
| Rv1991A | mazE6 | 2234742 | C150G | Ile50Met | 0.998 | - |
| Rv2009 | vapB15 | 2258035 | T6C | Tyr2Tyr | 4.42E-05 | 4.692(2.235-9.853) |
| Rv2009 | vapB15 | 2258096 | C67T | Arg23Trp | 0.267 | 0.599(0.242-1.48) |
| Rv2009 | vapB15 | 2258166 | T137C | Leu46Ser | 0.719 | 1.265(0.353-4.531) |
| Rv2009 | vapB15 | 2258266 | G237A | Glu79Glu | 0.014 | 2.765(1.231-6.207) |
| Rv2063 | mazE7 | 2321043 | G213T | Thr71Thr | 0.396 | 0.618(0.204-1.878) |
| Rv2104c | vapB37 | 2364533 | G249T | Gly83Gly | 0.096 | 3.607(0.798-16.312) |
| Rv2104c | vapB37 | 2364577 | G205C | Ala69Pro | 0.485 | 1.743(0.366-8.305) |
| Rv2104c | vapB37 | 2364766 | A16T | Thr6Ser | 0.311 | 0.58(0.203-1.661) |
| Rv2142c | parE2 | 2402223 | G288C | Glu96Asp | 0.998 | - |
| Rv2142c | parE2 | 2402439 | C72T | Asp24Asp | 0.156 | 1.265(0.914-1.749) |
| Rv2142c | parE2 | 2402463 | C48G | Phe16Leu | 7.44E-06 | 6.461(2.857-14.612) |
| Rv2142A | parD2 | 2402527 | A196G | Ile66Val | 0.011 | 3.531(1.337-9.327) |
| Rv2142A | parD2 | 2402684 | A39G | Ala13Ala | 0.200 | 3.17(0.542-18.541) |
| Rv2274c | mazF8 | 2546543 | G263A | Arg88Gln | 0.985 | 1.012(0.301-3.405) |
| Rv2274c | mazF8 | 2546601 | A205G | Ile69Val | 0.815 | 0.822(0.16-4.228) |
| Rv2274c | mazF8 | 2546684 | G122T | Gly41Val | 0.982 | - |
| Rv2274c | mazF8 | 2546698 | A108T | Val36Val | 0.186 | 1.963(0.722-5.333) |
| Rv2274c | mazF8 | 2546707 | A99G | Ile33Met | 0.427 | 0.524(0.106-2.583) |
| Rv2274c | mazF8 | 2546709 | A97G | Ile33Val | 4.77E-07 | 21.31(6.478-70.103) |
| Rv2274c | mazF8 | 2546710 | C96A | His32Gln | 0.246 | 0.522(0.174-1.567) |
| Rv2274c | mazF8 | 2546803 | G3A | Met1? | 0.799 | 0.887(0.352-2.234) |
| Rv2274A | mazE8 | 2546842 | T246C | Cys82Cys | 0.998 | - |
| Rv2493 | vapB38 | 2808218 | G136A | Asp46Asn | 0.997 | - |
| Rv2526 | vapB17 | 2851303 | G213C | Glu71Asp | 0.033 | 3.93(1.119-13.795) |
| Rv2530A | vapB39 | 2854891 | A17G | Gln6Arg | 0.528 | 2.205(0.19-25.613) |
| Rv2545 | vapB18 | 2867913 | T131G | Leu44Arg | 0.882 | 0.945(0.446-2.001) |
| Rv2547 | vapB19 | 2868606 | A1G | Met1? | 0.989 | - |
| Rv2547 | vapB19 | 2868659 | C54G | Ala18Ala | 0.700 | 0.96(0.782-1.179) |
| Rv2547 | vapB19 | 2868716 | C111T | Ala37Ala | 0.484 | 1.371(0.567-3.319) |
| Rv2547 | vapB19 | 2868769 | G164A | Gly55Asp | 1.000 | - |
| Rv2547 | vapB19 | 2868774 | T169C | Trp57Arg | 0.715 | 1.11(0.635-1.939) |
| Rv2547 | vapB19 | 2868787 | A182G | Asp61Gly | 0.062 | 2.944(0.946-9.161) |
| Rv2547 | vapB19 | 2868793 | C188T | Thr63Ile | 0.818 | 0.914(0.425-1.964) |
| Rv2550c | vapB20 | 2870142 | G223T | Val75Leu | 0.429 | 0.714(0.311-1.643) |
| Rv2550c | vapB20 | 2870311 | A54C | Glu18Asp | 0.044 | 3.111(1.033-9.375) |
| Rv2595 | vapB40 | 2925530 | T39C | Ile13Ile | 0.995 | - |
| Rv2595 | vapB40 | 2925594 | G103A | Asp35Asn | 1.000 | - |
| Rv2601A | vapB41 | 2930254 | G185A | Gly62Asp | 0.014 | 0.512(0.299-0.875) |
| Rv2601A | vapB41 | 2930266 | G197A | Arg66His | 0.234 | 2.381(0.571-9.926) |
| Rv2653c | - | 2976587 | G323A | Ter108Ter | 0.989 | - |
| Rv2653c | - | 2976596 | G314A | Ser105Asn | 0.039 | 0.25(0.067-0.934) |
| Rv2653c | - | 2976598 | C312T | Asp104Asp | 0.717 | 1.254(0.369-4.257) |
| Rv2653c | - | 2976616 | C294G | His98Gln | 0.997 | - |
| Rv2653c | - | 2976638 | A272T | Lys91Met | 0.997 | - |
| Rv2653c | - | 2976654 | C256T | Leu86Phe | 0.978 | - |
| Rv2653c | - | 2976814 | C96T | Ala32Ala | 0.258 | 1.533(0.731-3.215) |
| Rv2653c | - | 2976830 | A80C | Gln27Pro | 0.122 | 1.465(0.903-2.376) |
| Rv2653c | - | 2976840 | G70A | Val24Ile | 0.265 | 0.516(0.161-1.651) |
| Rv2653c | - | 2976856 | C54A | Ala18Ala | 0.604 | 0.792(0.329-1.908) |
| Rv2654c | - | 2977033 | A202C | Thr68Pro | 0.177 | 2.146(0.708-6.509) |
| Rv2654c | - | 2977040 | C195T | Phe65Phe | 0.792 | 1.2(0.309-4.663) |
| Rv2654c | - | 2977083 | T152C | Val51Ala | 0.049 | 2.795(1.002-7.795) |
| Rv2654c | - | 2977157 | T78G | Ala26Ala | 0.004 | 0.118(0.027-0.505) |
| Rv2654c | - | 2977191 | C44T | Ala15Val | 0.256 | 1.989(0.607-6.517) |
| Rv2758c | vapB21 | 3070648 | G202A | Gly68Arg | 0.992 | - |
| Rv2760c | vapB42 | 3071291 | C246A | Tyr82* | 0.995 | - |
| Rv2760c | vapB42 | 3071308 | C229G | His77Asp | 0.994 | - |
| Rv2830c | vapB22 | 3137058 | C167T | Ala56Val | 0.280 | 0.822(0.576-1.173) |
| Rv2862A | vapB23 | 3174748 | T2C | Ile1? | 0.005 | 2.271(1.273-4.05) |
| Rv2865 | relF | 3177567 | G31A | Gly11Ser | 0.740 | 1.18(0.444-3.134) |
| Rv2865 | relF | 3177660 | G124A | Ala42Thr | 0.699 | 0.886(0.481-1.634) |
| Rv2866 | relG | 3177884 | C63A | Arg21Arg | 0.987 | - |
| Rv2871 | vapB43 | 3183165 | G28C | Glu10Gln | 0.005 | 6.967(1.816-26.73) |
| Rv2871 | vapB43 | 3183174 | C37T | Arg13Cys | 0.884 | 0.829(0.067-10.232) |
| Rv2871 | vapB43 | 3183210 | A73G | Thr25Ala | 0.079 | 1.975(0.923-4.227) |
| Rv3181c | - | 3550092 | G52T | Val18Phe | 0.722 | 1.153(0.526-2.528) |
| Rv3357 | relJ | 3770777 | G5A | Ser2Asn | 0.994 | - |
| Rv3358 | relK | 3771123 | C79T | Arg27Trp | 0.660 | 1.575(0.207-11.966) |
| Rv3358 | relK | 3771183 | G139A | Glu47Lys | 0.024 | 0.215(0.057-0.818) |
| Rv3358 | relK | 3771245 | G201T | Leu67Leu | 0.160 | 5.614(0.507-62.15) |
| Rv3358 | relK | 3771264 | G220A | Asp74Asn | 0.130 | 0.374(0.105-1.335) |
| Rv3385c | vapB46 | 3799821 | C123T | Asp41Asp | 0.446 | 2.056(0.322-13.142) |
| Rv3385c | vapB46 | 3799874 | G70A | Ala24Thr | 0.002 | 2.74(1.427-5.263) |
| Rv3407 | vapB47 | 3826316 | G65T | Arg22Leu | 0.998 | - |
| Rv3407 | vapB47 | 3826501 | C250T | Arg84Cys | 0.406 | 1.112(0.866-1.428) |
| Rv3697A | vapB48 | 4140319 | C145T | Arg49Cys | 0.930 | 0.894(0.074-10.813) |
| Rv3697A | vapB48 | 4140384 | G80A | Gly27Glu | 0.083 | 7.286(0.769-69.067) |
| Rv3697A | vapB48 | 4140443 | C21T | Leu7Leu | 0.998 | - |

OR, odds ratio; CI, confidence interval.

**Supplementary Table 6** Generalized linear mixed model analysis on clustered and non-clustered isolates in the lineage2 cohort.

| **Rv number** | **Gene** | **Position** | **SNP** | **Amino acid changes** | **P value** | **OR (95%CI)** |
| --- | --- | --- | --- | --- | --- | --- |
| Rv0239 | vapB24 | 289179 | A76C | Thr26Pro | 0.002 | 28.078(3.303-238.727) |
| Rv0298 | - | 363375 | C124A | Leu42Met | 0.238 | 1.761(0.689-4.504) |
| Rv0298 | - | 363464 | G213A | Arg71Arg | 0.123 | 3.494(0.714-17.102) |
| Rv0456A | mazF1 | 547158 | A200G | Glu67Gly | 1.000 | - |
| Rv0608 | vapB28 | 703306 | C63T | Thr21Thr | 1.000 | - |
| Rv0657c | vapB6 | 753589 | A29C | Asp10Ala | 0.991 | 0.994(0.354-2.792) |
| Rv0659c | mazF2 | 754909 | C85T | Arg29Cys | 0.009 | 3.967(1.405-11.201) |
| Rv0660c | mazE2 | 755094 | G132C | Gln44His | 0.169 | 0.48(0.168-1.367) |
| Rv0662c | vapB7 | 755947 | G77A | Arg26His | 0.481 | 0.673(0.224-2.024) |
| Rv0664 | vapB8 | 758548 | G17T | Cys6Phe | 0.648 | 0.799(0.305-2.093) |
| Rv1241 | vapB33 | 1384315 | G38A | Arg13Gln | 0.072 | 2.275(0.928-5.576) |
| Rv1494 | mazE4 | 1686299 | T29G | Ile10Ser | 0.014 | 0.36(0.159-0.817) |
| Rv1839c | vapB13 | 2087847 | C66T | Arg22Arg | 0.241 | 2.47(0.544-11.211) |
| Rv1955 | higB | 2201720 | T2C | Val1? | 0.301 | 0.716(0.38-1.349) |
| Rv1955 | higB | 2201808 | C90G | Asp30Glu | 0.956 | 1.016(0.573-1.801) |
| Rv1956 | higA | 2202472 | C335T | Ser112Phe | 1.000 | - |
| Rv1956 | higA | 2202491 | A354G | Ala118Ala | 0.419 | 0.534(0.117-2.446) |
| Rv1956 | higA | 2202500 | C363T | His121His | 0.103 | 5.977(0.699-51.121) |
| Rv1959c | parE1 | 2203953 | C25G | Pro9Ala | 1.32E-06 | 26.18(6.971-98.317) |
| Rv1991A | mazE6 | 2234736 | G156A | Arg52Arg | 1.21E-04 | 11.822(3.356-41.65) |
| Rv2009 | vapB15 | 2258096 | C67T | Arg23Trp | 0.336 | 0.644(0.263-1.579) |
| Rv2009 | vapB15 | 2258166 | T137C | Leu46Ser | 0.074 | 3.228(0.89-11.708) |
| Rv2104c | vapB37 | 2364766 | A16T | Thr6Ser | 0.733 | 1.203(0.415-3.491) |
| Rv2142A | parD2 | 2402684 | A39G | Ala13Ala | 0.113 | 6.973(0.632-76.938) |
| Rv2274c | mazF8 | 2546601 | A205G | Ile69Val | 0.844 | 0.847(0.162-4.427) |
| Rv2274c | mazF8 | 2546684 | G122T | Gly41Val | 0.984 | - |
| Rv2274c | mazF8 | 2546707 | A99G | Ile33Met | 0.178 | 0.23(0.027-1.949) |
| Rv2274c | mazF8 | 2546710 | C96A | His32Gln | 0.464 | 0.662(0.219-1.999) |
| Rv2545 | vapB18 | 2867913 | T131G | Leu44Arg | 0.978 | 1.01(0.483-2.112) |
| Rv2547 | vapB19 | 2868787 | A182G | Asp61Gly | 0.111 | 2.506(0.81-7.757) |
| Rv2595 | vapB40 | 2925530 | T39C | Ile13Ile | 0.999 | - |
| Rv2653c | - | 2976598 | C312T | Asp104Asp | 0.637 | 1.337(0.401-4.458) |
| Rv2653c | - | 2976654 | C256T | Leu86Phe | 0.981 | - |
| Rv2653c | - | 2976856 | C54A | Ala18Ala | 0.801 | 0.858(0.259-2.838) |
| Rv2654c | - | 2977040 | C195T | Phe65Phe | 0.821 | 1.169(0.301-4.538) |
| Rv2654c | - | 2977157 | T78G | Ala26Ala | 0.004 | 0.118(0.027-0.503) |
| Rv2760c | vapB42 | 3071308 | C229G | His77Asp | 0.999 | - |
| Rv2830c | vapB22 | 3137058 | C167T | Ala56Val | 0.984 | - |
| Rv2862A | vapB23 | 3174748 | T2C | Ile1? | 0.003 | 2.582(1.393-4.784) |
| Rv2865 | relF | 3177660 | G124A | Ala42Thr | 0.680 | 0.877(0.472-1.632) |
| Rv2866 | relG | 3177884 | C63A | Arg21Arg | 0.988 | - |
| Rv3181c | - | 3550092 | G52T | Val18Phe | 0.992 | 1.004(0.449-2.245) |
| Rv3358 | relK | 3771264 | G220A | Asp74Asn | 0.307 | 0.52(0.148-1.826) |
| Rv3407 | vapB47 | 3826501 | C250T | Arg84Cys | 0.450 | 1.104(0.854-1.426) |

OR, odds ratio; CI, confidence interval.

**Supplementary Table 7** Generalized linear mixed model analysis on clustered and non-clustered isolates in the lineage4 cohort.

| **Rv number** | **Gene** | **Position** | **SNP** | **Amino acid changes** | **P value** | **OR (95%CI)** |
| --- | --- | --- | --- | --- | --- | --- |
| Rv0064A | vapB1 | 71622 | G34A | Asp12Asn | 0.005 | 2.806(1.371-5.741) |
| Rv0298 | - | 363450 | T199G | Tyr67Asp | 0.990 | - |
| Rv0300 | vapB2 | 363861 | C36T | Asp12Asp | 0.944 | 0.944(0.19-4.691) |
| Rv0300 | vapB2 | 363996 | T171C | Gly57Gly | 0.024 | 2.818(1.144-6.94) |
| Rv0550c | vapB3 | 640862 | G43A | Val15Met | 0.213 | 0.374(0.079-1.76) |
| Rv0596c | vapB4 | 695231 | G255A | Trp85* | 0.502 | 0.603(0.138-2.642) |
| Rv0608 | vapB28 | 703440 | G197A | Arg66His | 0.094 | 0.229(0.041-1.284) |
| Rv0616A | vapB29 | 710907 | C126G | Arg42Arg | 0.990 | - |
| Rv0626 | vapB5 | 718144 | G120A | Pro40Pro | 0.762 | 1.389(0.166-11.621) |
| Rv0626 | vapB5 | 718231 | C207T | Asn69Asn | 0.801 | 0.906(0.422-1.945) |
| Rv0657c | vapB6 | 753536 | G82A | Ala28Thr | 0.721 | 1.206(0.432-3.365) |
| Rv0657c | vapB6 | 753562 | T56C | Ile19Thr | 0.581 | 1.128(0.735-1.732) |
| Rv0660c | mazE2 | 755069 | C157G | Pro53Ala | 0.706 | 0.786(0.224-2.751) |
| Rv0660c | mazE2 | 755122 | G104A | Arg35His | 0.006 | 3.115(1.378-7.038) |
| Rv0662c | vapB7 | 755873 | C151T | Arg51Cys | 0.326 | 0.712(0.361-1.402) |
| Rv0748 | vapB31 | 840982 | C36T | Leu12Leu | 0.100 | 5.363(0.725-39.696) |
| Rv1103c | mazE3 | 1231236 | G56A | Gly19Asp | 0.302 | 2.011(0.534-7.576) |
| Rv1113 | vapB32 | 1239471 | C56T | Thr19Ile | 5.80E-06 | 0.266(0.15-0.471) |
| Rv1246c | relE | 1388827 | A152T | Asn51Ile | 0.315 | 0.319(0.034-2.964) |
| Rv1247c | relB | 1389019 | T226A | Phe76Ile | 0.078 | 3.923(0.858-17.949) |
| Rv1398c | vapB10 | 1574765 | G3A | Met1? | 0.462 | 0.62(0.174-2.214) |
| Rv1494 | mazE4 | 1686352 | C82T | Arg28Cys | 0.703 | 0.814(0.282-2.347) |
| Rv1494 | mazE4 | 1686516 | C246T | Ala82Ala | 0.821 | 1.223(0.213-7.003) |
| Rv1560 | vapB11 | 1764808 | C54T | Ala18Ala | 6.78E-06 | 10.003(3.669-27.271) |
| Rv1740 | vapB34 | 1967844 | C140A | Ala47Glu | 0.118 | 0.7(0.448-1.095) |
| Rv1740 | vapB34 | 1967897 | G193C | Glu65Gln | 0.596 | 1.309(0.483-3.543) |
| Rv1942c | mazF5 | 2194950 | A24C | Gly8Gly | 0.721 | 1.148(0.537-2.457) |
| Rv1943c | mazE5 | 2195004 | T344C | Leu115Pro | 0.005 | 19.158(2.435-150.733) |
| Rv1943c | mazE5 | 2195025 | A323G | Glu108Gly | 0.997 | - |
| Rv1943c | mazE5 | 2195321 | G27A | Thr9Thr | 0.995 | - |
| Rv1952 | vapB14 | 2200754 | A29G | Lys10Arg | 0.002 | 2.219(1.357-3.63) |
| Rv1955 | higB | 2201853 | G135A | Gln45Gln | 0.082 | 2.116(0.909-4.925) |
| Rv1956 | higA | 2202582 | G445T | Ala149Ser | 0.993 | 1.011(0.078-13.093) |
| Rv1959c | parE1 | 2203869 | G109A | Glu37Lys | 0.101 | 5.772(0.712-46.759) |
| Rv1959c | parE1 | 2203875 | C103T | Leu35Leu | 0.406 | 1.28(0.715-2.29) |
| Rv1959c | parE1 | 2203890 | C88T | Gln30* | 5.10E-13 | 10.267(5.457-19.315) |
| Rv1960c | parD1 | 2204092 | C134T | Thr45Ile | 0.001 | 4.27(1.862-9.791) |
| Rv1960c | parD1 | 2204203 | T23C | Val8Ala | 1.000 | - |
| Rv1962A | vapB35 | 2205283 | G267A | Arg89Arg | 0.522 | 0.666(0.193-2.306) |
| Rv1962A | vapB35 | 2205451 | G99A | Lys33Lys | 0.328 | 0.837(0.586-1.195) |
| Rv1962A | vapB35 | 2205511 | G39A | Thr13Thr | 0.726 | 0.922(0.585-1.452) |
| Rv1991A | mazE6 | 2234742 | C150G | Ile50Met | 0.997 | - |
| Rv2009 | vapB15 | 2258035 | T6C | Tyr2Tyr | 0.040 | 2.218(1.036-4.753) |
| Rv2009 | vapB15 | 2258266 | G237A | Glu79Glu | 0.008 | 2.993(1.333-6.721) |
| Rv2063 | mazE7 | 2321043 | G213T | Thr71Thr | 0.329 | 0.572(0.187-1.755) |
| Rv2104c | vapB37 | 2364533 | G249T | Gly83Gly | 0.006 | 8.238(1.804-37.608) |
| Rv2104c | vapB37 | 2364577 | G205C | Ala69Pro | 0.557 | 1.66(0.306-9.003) |
| Rv2142c | parE2 | 2402439 | C72T | Asp24Asp | 0.333 | 1.176(0.847-1.632) |
| Rv2142c | parE2 | 2402463 | C48G | Phe16Leu | 0.005 | 3.301(1.438-7.575) |
| Rv2274c | mazF8 | 2546543 | G263A | Arg88Gln | 0.923 | 1.062(0.315-3.578) |
| Rv2274c | mazF8 | 2546709 | A97G | Ile33Val | 0.001 | 29.592(3.984-219.821) |
| Rv2274c | mazF8 | 2546803 | G3A | Met1? | 0.523 | 1.359(0.53-3.481) |
| Rv2274A | mazE8 | 2546842 | T246C | Cys82Cys | 0.996 | - |
| Rv2493 | vapB38 | 2808218 | G136A | Asp46Asn | 0.995 | - |
| Rv2526 | vapB17 | 2851303 | G213C | Glu71Asp | 0.037 | 3.828(1.087-13.487) |
| Rv2547 | vapB19 | 2868606 | A1G | Met1? | 0.991 | - |
| Rv2547 | vapB19 | 2868659 | C54G | Ala18Ala | 0.335 | 1.116(0.893-1.394) |
| Rv2547 | vapB19 | 2868774 | T169C | Trp57Arg | 0.058 | 0.572(0.32-1.02) |
| Rv2547 | vapB19 | 2868793 | C188T | Thr63Ile | 0.948 | 0.975(0.459-2.073) |
| Rv2550c | vapB20 | 2870142 | G223T | Val75Leu | 0.399 | 0.698(0.303-1.608) |
| Rv2550c | vapB20 | 2870311 | A54C | Glu18Asp | 0.020 | 3.792(1.228-11.705) |
| Rv2601A | vapB41 | 2930254 | G185A | Gly62Asp | 0.001 | 0.387(0.223-0.671) |
| Rv2653c | - | 2976587 | G323A | Ter108Ter | 0.990 | - |
| Rv2653c | - | 2976596 | G314A | Ser105Asn | 0.011 | 0.215(0.066-0.704) |
| Rv2653c | - | 2976814 | C96T | Ala32Ala | 0.267 | 1.521(0.725-3.188) |
| Rv2653c | - | 2976830 | A80C | Gln27Pro | 0.146 | 1.467(0.875-2.46) |
| Rv2653c | - | 2976840 | G70A | Val24Ile | 0.118 | 0.39(0.119-1.272) |
| Rv2654c | - | 2977033 | A202C | Thr68Pro | 0.101 | 2.511(0.836-7.542) |
| Rv2654c | - | 2977083 | T152C | Val51Ala | 0.071 | 2.575(0.922-7.188) |
| Rv2654c | - | 2977191 | C44T | Ala15Val | 0.205 | 2.154(0.657-7.059) |
| Rv2830c | vapB22 | 3137058 | C167T | Ala56Val | 0.093 | 0.723(0.495-1.056) |
| Rv2865 | relF | 3177567 | G31A | Gly11Ser | 0.759 | 1.165(0.44-3.086) |
| Rv2866 | relG | 3177884 | C63A | Arg21Arg | 0.996 | - |
| Rv2871 | vapB43 | 3183165 | G28C | Glu10Gln | 0.004 | 7.272(1.882-28.103) |
| Rv3358 | relK | 3771183 | G139A | Glu47Lys | 0.506 | 0.64(0.172-2.381) |
| Rv3385c | vapB46 | 3799874 | G70A | Ala24Thr | 0.041 | 2.014(1.03-3.937) |
| Rv3697A | vapB48 | 4140319 | C145T | Arg49Cys | 0.895 | 0.841(0.064-10.99) |
| Rv3697A | vapB48 | 4140384 | G80A | Gly27Glu | 0.113 | 6.028(0.654-55.575) |
| Rv3697A | vapB48 | 4140443 | C21T | Leu7Leu | 0.997 | - |

OR, odds ratio; CI, confidence interval.

**Supplementary Table 8** Generalized linear mixed model analysis on cross-country transmission clades.

| **Rv number** | **Gene** | **Position** | **SNP** | **Amino acid changes** | **P value** | **OR (95%CI)** |
| --- | --- | --- | --- | --- | --- | --- |
| Rv0064A | vapB1 | 71622 | G34A | Asp12Asn | 0.311 | 0.473(0.111-2.012) |
| Rv0239 | vapB24 | 289179 | A76C | Thr26Pro | 0.999 | - |
| Rv0239 | vapB24 | 289300 | C197T | Ser66Phe | 1.000 | - |
| Rv0298 | - | 363375 | C124A | Leu42Met | 0.995 | - |
| Rv0298 | - | 363464 | G213A | Arg71Arg | 0.999 | - |
| Rv0298 | - | 363467 | G216A | Ala72Ala | 0.838 | 0.928(0.454-1.899) |
| Rv0300 | vapB2 | 363996 | T171C | Gly57Gly | 0.999 | - |
| Rv0456A | mazF1 | 547158 | A200G | Glu67Gly | 1.000 | - |
| Rv0596c | vapB4 | 695231 | G255A | Trp85* | 0.998 | - |
| Rv0599c | vapB27 | 697606 | C195T | Asp65Asp | 1.000 | - |
| Rv0608 | vapB28 | 703306 | C63T | Thr21Thr | 1.000 | - |
| Rv0616A | vapB29 | 710907 | C126G | Arg42Arg | 0.999 | - |
| Rv0616A | vapB29 | 710997 | G216A | Glu72Glu | 1.000 | - |
| Rv0626 | vapB5 | 718231 | C207T | Asn69Asn | 0.999 | - |
| Rv0657c | vapB6 | 753536 | G82A | Ala28Thr | 0.999 | - |
| Rv0657c | vapB6 | 753562 | T56C | Ile19Thr | 0.696 | 1.168(0.537-2.54) |
| Rv0657c | vapB6 | 753589 | A29C | Asp10Ala | 3.93E-05 | 11.28(3.554-35.803) |
| Rv0659c | mazF2 | 754909 | C85T | Arg29Cys | 0.999 | - |
| Rv0660c | mazE2 | 755094 | G132C | Gln44His | 0.999 | - |
| Rv0660c | mazE2 | 755122 | G104A | Arg35His | 5.40E-05 | 0.117(0.041-0.331) |
| Rv0662c | vapB7 | 755873 | C151T | Arg51Cys | 0.921 | 0.953(0.366-2.482) |
| Rv0664 | vapB8 | 758548 | G17T | Cys6Phe | 0.999 | - |
| Rv0748 | vapB31 | 841058 | T112C | Phe38Leu | 0.004 | 9.796(2.07-46.371) |
| Rv1103c | mazE3 | 1231236 | G56A | Gly19Asp | 0.999 | - |
| Rv1113 | vapB32 | 1239471 | C56T | Thr19Ile | 0.999 | - |
| Rv1241 | vapB33 | 1384315 | G38A | Arg13Gln | 1.000 | - |
| Rv1246c | relE | 1388827 | A152T | Asn51Ile | 0.028 | 0.168(0.034-0.825) |
| Rv1247c | relB | 1389019 | T226A | Phe76Ile | 0.999 | - |
| Rv1494 | mazE4 | 1686299 | T29G | Ile10Ser | 0.296 | 0.446(0.098-2.025) |
| Rv1560 | vapB11 | 1764808 | C54T | Ala18Ala | 0.998 | - |
| Rv1740 | vapB34 | 1967783 | A79G | Thr27Ala | 1.000 | - |
| Rv1740 | vapB34 | 1967844 | C140A | Ala47Glu | 0.292 | 0.572(0.202-1.618) |
| Rv1740 | vapB34 | 1967897 | G193C | Glu65Gln | 0.499 | 1.715(0.359-8.185) |
| Rv1942c | mazF5 | 2194950 | A24C | Gly8Gly | 0.576 | 1.394(0.435-4.466) |
| Rv1943c | mazE5 | 2195004 | T344C | Leu115Pro | 1.000 | - |
| Rv1943c | mazE5 | 2195025 | A323G | Glu108Gly | 0.198 | 2.046(0.688-6.083) |
| Rv1943c | mazE5 | 2195321 | G27A | Thr9Thr | 0.776 | 0.857(0.297-2.474) |
| Rv1952 | vapB14 | 2200754 | A29G | Lys10Arg | 0.153 | 0.471(0.167-1.323) |
| Rv1955 | higB | 2201720 | T2C | Val1? | 0.151 | 1.934(0.786-4.759) |
| Rv1955 | higB | 2201808 | C90G | Asp30Glu | 0.995 | - |
| Rv1955 | higB | 2201853 | G135A | Gln45Gln | 0.999 | - |
| Rv1956 | higA | 2202472 | C335T | Ser112Phe | 1.000 | - |
| Rv1956 | higA | 2202491 | A354G | Ala118Ala | 1.000 | - |
| Rv1956 | higA | 2202500 | C363T | His121His | 0.164 | 0.134(0.008-2.273) |
| Rv1956 | higA | 2202582 | G445T | Ala149Ser | 0.533 | 1.157(0.731-1.832) |
| Rv1959c | parE1 | 2203869 | G109A | Glu37Lys | 0.998 | - |
| Rv1959c | parE1 | 2203875 | C103T | Leu35Leu | 0.019 | 0.226(0.065-0.786) |
| Rv1959c | parE1 | 2203890 | C88T | Gln30* | 0.999 | - |
| Rv1959c | parE1 | 2203953 | C25G | Pro9Ala | 1.000 | - |
| Rv1960c | parD1 | 2204019 | C207T | Asp69Asp | 1.000 | - |
| Rv1960c | parD1 | 2204092 | C134T | Thr45Ile | 1.07E-07 | 7.737(3.638-16.455) |
| Rv1960c | parD1 | 2204203 | T23C | Val8Ala | 0.999 | - |
| Rv1962A | vapB35 | 2205283 | G267A | Arg89Arg | 0.998 | - |
| Rv1962A | vapB35 | 2205451 | G99A | Lys33Lys | 0.149 | 1.514(0.861-2.662) |
| Rv1962A | vapB35 | 2205511 | G39A | Thr13Thr | 0.999 | - |
| Rv1991A | mazE6 | 2234736 | G156A | Arg52Arg | 1.000 | - |
| Rv2009 | vapB15 | 2258035 | T6C | Tyr2Tyr | 0.999 | - |
| Rv2009 | vapB15 | 2258266 | G237A | Glu79Glu | 0.999 | - |
| Rv2063 | mazE7 | 2321043 | G213T | Thr71Thr | 0.117 | 4.221(0.698-25.53) |
| Rv2104c | vapB37 | 2364533 | G249T | Gly83Gly | 0.999 | - |
| Rv2104c | vapB37 | 2364577 | G205C | Ala69Pro | 2.67E-06 | 22.874(6.193-84.482) |
| Rv2142c | parE2 | 2402223 | G288C | Glu96Asp | 1.000 | - |
| Rv2142c | parE2 | 2402439 | C72T | Asp24Asp | 0.367 | 0.74(0.384-1.424) |
| Rv2142c | parE2 | 2402463 | C48G | Phe16Leu | 0.999 | - |
| Rv2142A | parD2 | 2402527 | A196G | Ile66Val | 1.000 | - |
| Rv2142A | parD2 | 2402684 | A39G | Ala13Ala | 1.000 | - |
| Rv2274c | mazF8 | 2546684 | G122T | Gly41Val | 1.000 | - |
| Rv2274c | mazF8 | 2546707 | A99G | Ile33Met | 1.000 | - |
| Rv2274c | mazF8 | 2546709 | A97G | Ile33Val | 0.998 | - |
| Rv2274c | mazF8 | 2546803 | G3A | Met1? | 0.550 | 1.47(0.415-5.201) |
| Rv2526 | vapB17 | 2851303 | G213C | Glu71Asp | 0.999 | - |
| Rv2530A | vapB39 | 2854891 | A17G | Gln6Arg | 0.189 | 5.202(0.445-60.874) |
| Rv2545 | vapB18 | 2867913 | T131G | Leu44Arg | 0.999 | - |
| Rv2547 | vapB19 | 2868606 | A1G | Met1? | 0.999 | - |
| Rv2547 | vapB19 | 2868659 | C54G | Ala18Ala | 1.88E-09 | 0.258(0.166-0.401) |
| Rv2547 | vapB19 | 2868716 | C111T | Ala37Ala | 0.999 | - |
| Rv2547 | vapB19 | 2868774 | T169C | Trp57Arg | 1.000 | - |
| Rv2547 | vapB19 | 2868793 | C188T | Thr63Ile | 0.001 | 5.333(1.945-14.626) |
| Rv2550c | vapB20 | 2870142 | G223T | Val75Leu | 0.999 | - |
| Rv2601A | vapB41 | 2930254 | G185A | Gly62Asp | 0.524 | 0.711(0.249-2.03) |
| Rv2653c | - | 2976616 | C294G | His98Gln | 1.000 | - |
| Rv2653c | - | 2976814 | C96T | Ala32Ala | 1.000 | - |
| Rv2653c | - | 2976830 | A80C | Gln27Pro | 1.12E-09 | 5.447(3.157-9.398) |
| Rv2653c | - | 2976840 | G70A | Val24Ile | 1.000 | - |
| Rv2653c | - | 2976856 | C54A | Ala18Ala | 1.000 | - |
| Rv2654c | - | 2977033 | A202C | Thr68Pro | 0.999 | - |
| Rv2654c | - | 2977083 | T152C | Val51Ala | 0.999 | - |
| Rv2654c | - | 2977191 | C44T | Ala15Val | 0.999 | - |
| Rv2830c | vapB22 | 3137058 | C167T | Ala56Val | 0.012 | 3.691(1.332-10.231) |
| Rv2862A | vapB23 | 3174748 | T2C | Ile1? | 0.786 | 1.168(0.381-3.581) |
| Rv2865 | relF | 3177567 | G31A | Gly11Ser | 0.999 | - |
| Rv2865 | relF | 3177660 | G124A | Ala42Thr | 0.238 | 0.404(0.09-1.821) |
| Rv2871 | vapB43 | 3183174 | C37T | Arg13Cys | 0.342 | 0.283(0.021-3.834) |
| Rv2871 | vapB43 | 3183210 | A73G | Thr25Ala | 1.000 | - |
| Rv3181c | - | 3550092 | G52T | Val18Phe | 0.621 | 1.465(0.323-6.647) |
| Rv3358 | relK | 3771123 | C79T | Arg27Trp | 1.000 | - |
| Rv3385c | vapB46 | 3799821 | C123T | Asp41Asp | 1.000 | - |
| Rv3385c | vapB46 | 3799874 | G70A | Ala24Thr | 0.999 | - |
| Rv3407 | vapB47 | 3826316 | G65T | Arg22Leu | 0.733 | 1.5(0.146-15.451) |
| Rv3407 | vapB47 | 3826501 | C250T | Arg84Cys | 0.003 | 0.526(0.344-0.805) |

OR, odds ratio; CI, confidence interval.

**Supplementary Table 9** Generalized linear mixed model analysis on cross-regional transmission clades.

| **Rv number** | **Gene** | **Position** | **SNP** | **Amino acid changes** | **P value** | **OR (95%CI)** |
| --- | --- | --- | --- | --- | --- | --- |
| Rv0064A | vapB1 | 71622 | G34A | Asp12Asn | 0.999 | - |
| Rv0239 | vapB24 | 289179 | A76C | Thr26Pro | 0.999 | - |
| Rv0239 | vapB24 | 289300 | C197T | Ser66Phe | 1.000 | - |
| Rv0298 | - | 363375 | C124A | Leu42Met | 0.995 | - |
| Rv0298 | - | 363464 | G213A | Arg71Arg | 0.999 | - |
| Rv0298 | - | 363467 | G216A | Ala72Ala | 0.515 | 1.295(0.595-2.821) |
| Rv0300 | vapB2 | 363996 | T171C | Gly57Gly | 0.999 | - |
| Rv0456A | mazF1 | 547158 | A200G | Glu67Gly | 1.000 | - |
| Rv0596c | vapB4 | 695231 | G255A | Trp85* | 0.998 | - |
| Rv0599c | vapB27 | 697606 | C195T | Asp65Asp | 1.000 | - |
| Rv0608 | vapB28 | 703306 | C63T | Thr21Thr | 1.000 | - |
| Rv0616A | vapB29 | 710907 | C126G | Arg42Arg | 0.999 | - |
| Rv0616A | vapB29 | 710997 | G216A | Glu72Glu | 1.000 | - |
| Rv0626 | vapB5 | 718231 | C207T | Asn69Asn | 0.999 | - |
| Rv0657c | vapB6 | 753536 | G82A | Ala28Thr | 0.999 | - |
| Rv0657c | vapB6 | 753562 | T56C | Ile19Thr | 0.431 | 0.658(0.232-1.865) |
| Rv0657c | vapB6 | 753589 | A29C | Asp10Ala | 3.33E-05 | 11.542(3.636-36.643) |
| Rv0659c | mazF2 | 754909 | C85T | Arg29Cys | 0.998 | - |
| Rv0660c | mazE2 | 755094 | G132C | Gln44His | 0.998 | - |
| Rv0660c | mazE2 | 755122 | G104A | Arg35His | 5.42E-05 | 0.117(0.041-0.332) |
| Rv0662c | vapB7 | 755873 | C151T | Arg51Cys | 0.755 | 1.166(0.446-3.048) |
| Rv0664 | vapB8 | 758548 | G17T | Cys6Phe | 0.998 | - |
| Rv0748 | vapB31 | 841058 | T112C | Phe38Leu | 0.012 | 7.652(1.574-37.208) |
| Rv1103c | mazE3 | 1231236 | G56A | Gly19Asp | 0.999 | - |
| Rv1113 | vapB32 | 1239471 | C56T | Thr19Ile | 0.999 | - |
| Rv1241 | vapB33 | 1384315 | G38A | Arg13Gln | 1.000 | - |
| Rv1246c | relE | 1388827 | A152T | Asn51Ile | 0.254 | 0.364(0.064-2.066) |
| Rv1247c | relB | 1389019 | T226A | Phe76Ile | 0.999 | - |
| Rv1494 | mazE4 | 1686299 | T29G | Ile10Ser | 0.296 | 0.446(0.098-2.025) |
| Rv1560 | vapB11 | 1764808 | C54T | Ala18Ala | 0.998 | - |
| Rv1740 | vapB34 | 1967783 | A79G | Thr27Ala | 1.000 | - |
| Rv1740 | vapB34 | 1967844 | C140A | Ala47Glu | 0.502 | 0.699(0.246-1.986) |
| Rv1740 | vapB34 | 1967897 | G193C | Glu65Gln | 0.353 | 2.098(0.439-10.037) |
| Rv1942c | mazF5 | 2194950 | A24C | Gly8Gly | 0.396 | 1.662(0.514-5.377) |
| Rv1943c | mazE5 | 2195004 | T344C | Leu115Pro | 0.998 | - |
| Rv1943c | mazE5 | 2195025 | A323G | Glu108Gly | 0.198 | 2.045(0.688-6.082) |
| Rv1943c | mazE5 | 2195321 | G27A | Thr9Thr | 0.930 | 1.049(0.362-3.037) |
| Rv1952 | vapB14 | 2200754 | A29G | Lys10Arg | 0.079 | 0.278(0.067-1.158) |
| Rv1955 | higB | 2201720 | T2C | Val1? | 0.138 | 1.978(0.803-4.869) |
| Rv1955 | higB | 2201808 | C90G | Asp30Glu | 0.995 | - |
| Rv1955 | higB | 2201853 | G135A | Gln45Gln | 1.000 | - |
| Rv1956 | higA | 2202472 | C335T | Ser112Phe | 1.000 | - |
| Rv1956 | higA | 2202491 | A354G | Ala118Ala | 0.999 | - |
| Rv1956 | higA | 2202500 | C363T | His121His | 0.162 | 0.133(0.008-2.255) |
| Rv1956 | higA | 2202582 | G445T | Ala149Ser | 0.492 | 1.188(0.727-1.94) |
| Rv1959c | parE1 | 2203869 | G109A | Glu37Lys | 0.999 | - |
| Rv1959c | parE1 | 2203875 | C103T | Leu35Leu | 0.326 | 0.489(0.118-2.037) |
| Rv1959c | parE1 | 2203890 | C88T | Gln30* | 0.999 | - |
| Rv1959c | parE1 | 2203953 | C25G | Pro9Ala | 1.000 | - |
| Rv1960c | parD1 | 2204019 | C207T | Asp69Asp | 1.000 | - |
| Rv1960c | parD1 | 2204092 | C134T | Thr45Ile | 1.09E-07 | 7.729(3.634-16.438) |
| Rv1960c | parD1 | 2204203 | T23C | Val8Ala | 0.999 | - |
| Rv1962A | vapB35 | 2205283 | G267A | Arg89Arg | 0.998 | - |
| Rv1962A | vapB35 | 2205451 | G99A | Lys33Lys | 0.125 | 1.593(0.879-2.889) |
| Rv1962A | vapB35 | 2205511 | G39A | Thr13Thr | 1.000 | - |
| Rv1991A | mazE6 | 2234736 | G156A | Arg52Arg | 0.999 | - |
| Rv2009 | vapB15 | 2258035 | T6C | Tyr2Tyr | 0.998 | - |
| Rv2009 | vapB15 | 2258266 | G237A | Glu79Glu | 0.999 | - |
| Rv2063 | mazE7 | 2321043 | G213T | Thr71Thr | 0.116 | 4.242(0.701-25.658) |
| Rv2104c | vapB37 | 2364533 | G249T | Gly83Gly | 0.998 | - |
| Rv2104c | vapB37 | 2364577 | G205C | Ala69Pro | 6.13E-07 | 27.966(7.551-103.573) |
| Rv2142c | parE2 | 2402223 | G288C | Glu96Asp | 1.000 | - |
| Rv2142c | parE2 | 2402439 | C72T | Asp24Asp | 0.024 | 0.308(0.111-0.856) |
| Rv2142c | parE2 | 2402463 | C48G | Phe16Leu | 0.999 | - |
| Rv2142A | parD2 | 2402527 | A196G | Ile66Val | 1.000 | - |
| Rv2142A | parD2 | 2402684 | A39G | Ala13Ala | 1.000 | - |
| Rv2274c | mazF8 | 2546684 | G122T | Gly41Val | 1.000 | - |
| Rv2274c | mazF8 | 2546707 | A99G | Ile33Met | 1.000 | - |
| Rv2274c | mazF8 | 2546709 | A97G | Ile33Val | 0.998 | - |
| Rv2274c | mazF8 | 2546803 | G3A | Met1? | 0.999 | - |
| Rv2526 | vapB17 | 2851303 | G213C | Glu71Asp | 0.997 | - |
| Rv2530A | vapB39 | 2854891 | A17G | Gln6Arg | 0.117 | 7.257(0.608-86.609) |
| Rv2545 | vapB18 | 2867913 | T131G | Leu44Arg | 0.999 | - |
| Rv2547 | vapB19 | 2868606 | A1G | Met1? | 0.999 | - |
| Rv2547 | vapB19 | 2868659 | C54G | Ala18Ala | 5.11E-07 | 0.316(0.201-0.495) |
| Rv2547 | vapB19 | 2868716 | C111T | Ala37Ala | 0.999 | - |
| Rv2547 | vapB19 | 2868774 | T169C | Trp57Arg | 1.000 | - |
| Rv2547 | vapB19 | 2868793 | C188T | Thr63Ile | 2.82E-04 | 6.527(2.372-17.963) |
| Rv2550c | vapB20 | 2870142 | G223T | Val75Leu | 0.998 | - |
| Rv2601A | vapB41 | 2930254 | G185A | Gly62Asp | 0.791 | 0.867(0.303-2.483) |
| Rv2653c | - | 2976616 | C294G | His98Gln | 1.000 | - |
| Rv2653c | - | 2976814 | C96T | Ala32Ala | 1.000 | - |
| Rv2653c | - | 2976830 | A80C | Gln27Pro | 5.36E-10 | 5.83(3.342-10.17) |
| Rv2653c | - | 2976840 | G70A | Val24Ile | 0.999 | - |
| Rv2653c | - | 2976856 | C54A | Ala18Ala | 0.999 | - |
| Rv2654c | - | 2977033 | A202C | Thr68Pro | 0.999 | - |
| Rv2654c | - | 2977083 | T152C | Val51Ala | 1.000 | - |
| Rv2654c | - | 2977191 | C44T | Ala15Val | 0.999 | - |
| Rv2830c | vapB22 | 3137058 | C167T | Ala56Val | 0.033 | 3.031(1.091-8.426) |
| Rv2862A | vapB23 | 3174748 | T2C | Ile1? | 0.752 | 1.198(0.39-3.677) |
| Rv2865 | relF | 3177567 | G31A | Gly11Ser | 0.999 | - |
| Rv2865 | relF | 3177660 | G124A | Ala42Thr | 0.238 | 0.404(0.09-1.821) |
| Rv2871 | vapB43 | 3183174 | C37T | Arg13Cys | 0.180 | 0.157(0.01-2.355) |
| Rv2871 | vapB43 | 3183210 | A73G | Thr25Ala | 1.000 | - |
| Rv3181c | - | 3550092 | G52T | Val18Phe | 0.600 | 1.498(0.33-6.798) |
| Rv3358 | relK | 3771123 | C79T | Arg27Trp | 1.000 | - |
| Rv3385c | vapB46 | 3799821 | C123T | Asp41Asp | 1.000 | - |
| Rv3385c | vapB46 | 3799874 | G70A | Ala24Thr | 0.998 | - |
| Rv3407 | vapB47 | 3826316 | G65T | Arg22Leu | 0.733 | 1.5(0.146-15.458) |
| Rv3407 | vapB47 | 3826501 | C250T | Arg84Cys | 0.002 | 0.515(0.336-0.788) |

OR, odds ratio; CI, confidence interval.

**Supplementary Table 10** The performance of various models for discriminating clustered isolates from non-clustered isolates in lineage2 cohort.

| **Parameters** | **Training set** | | **Test set** | |
| --- | --- | --- | --- | --- |
|  | **(n=3593, 1411 clustered isolates,** | | **(n=1540, 632 clustered isolates,** | |
|  | **2182 non-clustered isolates)** | | **908 non-clustered isolates)** | |
|  | **Random Forest** | **Gradient Boosted Classification Tree** | **Random Forest** | **Gradient Boosted Classification Tree** |
| Kappa | 0.402 | 0.397 | 0.376 | 0.406 |
| AUC | 0.749 | 0.752 | 0.723 | 0.723 |
| (95% CI) | (0.735, 0.763) | (0.738, 0.766) | (0.701, 0.745) | (0.701, 0.745) |
| Sensitivity | 0.485 | 0.486 | 0.456 | 0.497 |
| (95% CI) | (0.469, 0.501) | (0.47, 0.502) | (0.431, 0.481) | (0.472, 0.522) |
| Specificity | 0.892 | 0.887 | 0.898 | 0.887 |
| (95% CI) | (0.882, 0.902) | (0.877, 0.897) | (0.883, 0.913) | (0.871, 0.903) |
| PPV | 0.744 | 0.737 | 0.756 | 0.751 |
| (95% CI) | (0.73, 0.758) | (0.723, 0.751) | (0.735, 0.777) | (0.729, 0.773) |
| NPV | 0.728 | 0.726 | 0.703 | 0.72 |
| (95% CI) | (0.713, 0.743) | (0.711, 0.741) | (0.68, 0.726) | (0.698, 0.742) |
| PLR | 2.738 | 2.691 | 2.547 | 2.685 |
| (95% CI) | (2.711, 2.765) | (2.664, 2.718) | (2.501, 2.593) | (2.642, 2.728) |
| NIR | 0.365 | 0.372 | 0.393 | 0.372 |
| (95% CI) | (0.307, 0.423) | (0.315, 0.429) | (0.301, 0.485) | (0.282, 0.462) |
| Accuracy | 0.732 | 0.729 | 0.716 | 0.729 |
| (95% CI) | (0.718, 0.746) | (0.714, 0.744) | (0.693, 0.739) | (0.707, 0.751) |

AUC, area under the curve; PPV, positive predictive value; NPV, negative predictive value; PLR, positive likelihood ratio; NLR, negative likelihood ratio; CI, confidence.

**Supplementary Table 11** The performance of various models for discriminating clustered isolates from non-clustered isolates in lineage4 cohort.

| **Parameters** | **Training set**  **(n=4541, 2265 clustered isolates,**  **2276 non-clustered isolates)** | | **Test set**  **(n=1947, 980 clustered isolates,**  **967non-clustered isolates)** | |
| --- | --- | --- | --- | --- |
|  | **Random Forest** | **Gradient Boosted Classification Tree** | **Random Forest** | **Gradient Boosted Classification Tree** |
| Kappa | 0.447 | 0.382 | 0.355 | 0.331 |
| AUC | 0.802 | 0.768 | 0.748 | 0.723 |
| (95% CI) | (0.79, 0.814) | (0.756, 0.78) | (0.729, 0.767) | (0.703, 0.743) |
| Sensitivity | 0.781 | 0.801 | 0.736 | 0.777 |
| (95% CI) | (0.769, 0.793) | (0.789, 0.813) | (0.716, 0.756) | (0.759, 0.795) |
| Specificity | 0.666 | 0.581 | 0.619 | 0.554 |
| (95% CI) | (0.652, 0.68) | (0.567, 0.595) | (0.597, 0.641) | (0.532, 0.576) |
| PPV | 0.699 | 0.657 | 0.662 | 0.634 |
| (95% CI) | (0.686, 0.712) | (0.643, 0.671) | (0.641, 0.683) | (0.613, 0.655) |
| NPV | 0.754 | 0.744 | 0.698 | 0.714 |
| (95% CI) | (0.741, 0.767) | (0.731, 0.757) | (0.678, 0.718) | (0.694, 0.734) |
| PLR | 2.84 | 2.572 | 2.193 | 2.215 |
| (95% CI) | (2.82, 2.86) | (2.552, 2.592) | (2.157, 2.229) | (2.182, 2.248) |
| NIR | 0.352 | 0.389 | 0.456 | 0.451 |
| (95% CI) | (0.305, 0.399) | (0.346, 0.432) | (0.389, 0.523) | (0.387, 0.515) |
| Accuracy | 0.723 | 0.691 | 0.678 | 0.665 |
| (95% CI) | (0.71, 0.736) | (0.678, 0.704) | (0.657, 0.699) | (0.644, 0.686) |

AUC, area under the curve; PPV, positive predictive value; NPV, negative predictive value; PLR, positive likelihood ratio; NLR, negative likelihood ratio; CI, confidence.

**Supplementary Table 12** The performance of various models for discriminating cross-country from non-cross-country transmission clades.

| **Parameters** | **Training set**  **(n=5465, 483 cross_country isolates,**  **4982 non_cross_country isolates)** | | **Test set**  **(n=2343, 221 cross_country isolates,**  **2122 non_cross_country isolates)** | |
| --- | --- | --- | --- | --- |
|  | **Random Forest** | **Gradient Boosted Classification Tree** | **Random Forest** | **Gradient Boosted Classification Tree** |
| Kappa | 0.094 | 0.047 | 0.055 | 0.026 |
| AUC | 0.722 | 0.69 | 0.673 | 0.654 |
| (95% CI) | (0.71, 0.734) | (0.678, 0.702) | (0.654, 0.692) | (0.635, 0.673) |
| Sensitivity | 0.058 | 0.028 | 0.036 | 0.015 |
| (95% CI) | (0.052, 0.064) | (0.024, 0.032) | (0.028, 0.044) | (0.01, 0.02) |
| Specificity | 0.997 | 0.999 | 0.996 | 0.999 |
| (95% CI) | (0.996, 0.998) | (0.998, 1.0) | (0.993, 0.999) | (0.998, 1.0) |
| PPV | 0.683 | 0.737 | 0.5 | 0.6 |
| (95% CI) | (0.671, 0.695) | (0.725, 0.749) | (0.48, 0.52) | (0.58, 0.62) |
| NPV | 0.916 | 0.909 | 0.908 | 0.918 |
| (95% CI) | (0.909, 0.923) | (0.901, 0.917) | (0.896, 0.92) | (0.907, 0.929) |
| PLR | 8.141 | 8.107 | 5.462 | 7.306 |
| (95% CI) | (8.133, 8.149) | (8.098, 8.116) | (5.451, 5.473) | (7.295, 7.317) |
| NIR | 0.123 | 0.123 | 0.183 | 0.137 |
| (95% CI) | (0.082, 0.164) | (0.077, 0.169) | (0.137, 0.229) | (0.083, 0.191) |
| Accuracy | 0.914 | 0.909 | 0.906 | 0.917 |
| (95% CI) | (0.907, 0.921) | (0.901, 0.917) | (0.894, 0.918) | (0.906, 0.928) |

AUC, area under the curve; PPV, positive predictive value; NPV, negative predictive value; PLR, positive likelihood ratio; NLR, negative likelihood ratio; CI, confidence.

**Supplementary Table 13** The performance of various models for discriminating cross-regional from non-cross-regional transmission clades.

| Parameters | Training set  (n=5465, 449 cross_regional isolates,  5016 non_cross_regional isolates) | | Test set  (n=2343, 201 cross_regional isolates,  2142 non_cross_regional isolates) | |
| --- | --- | --- | --- | --- |
|  | Random Forest | Gradient Boosted Classification Tree | Random Forest | Gradient Boosted Classification Tree |
| Kappa | 0.085 | 0.06 | 0.059 | 0.084 |
| AUC | 0.732 | 0.693 | 0.667 | 0.705 |
| (95% CI) | (0.72, 0.744) | (0.681, 0.705) | (0.648, 0.686) | (0.687, 0.723) |
| Sensitivity | 0.051 | 0.036 | 0.035 | 0.05 |
| (95% CI) | (0.045, 0.057) | (0.031, 0.041) | (0.028, 0.042) | (0.041, 0.059) |
| Specificity | 0.998 | 0.998 | 0.999 | 0.999 |
| (95% CI) | (0.997, 0.999) | (0.997, 0.999) | (0.998, 1.0) | (0.998, 1.0) |
| PPV | 0.676 | 0.654 | 0.7 | 0.75 |
| (95% CI) | (0.664, 0.688) | (0.641, 0.667) | (0.681, 0.719) | (0.732, 0.768) |
| NPV | 0.922 | 0.917 | 0.917 | 0.926 |
| (95% CI) | (0.915, 0.929) | (0.91, 0.924) | (0.906, 0.928) | (0.915, 0.937) |
| PLR | 8.624 | 7.868 | 8.418 | 10.164 |
| (95% CI) | (8.617, 8.631) | (7.86, 7.876) | (8.406, 8.43) | (10.152, 10.176) |
| NIR | 0.116 | 0.127 | 0.119 | 0.098 |
| (95% CI) | (0.076, 0.156) | (0.088, 0.166) | (0.054, 0.184) | (0.026, 0.17) |
| Accuracy | 0.920 | 0.916 | 0.916 | 0.925 |
| (95% CI) | (0.913, 0.927) | (0.909, 0.923) | (0.905, 0.927) | (0.914, 0.936) |

AUC, area under the curve; PPV, positive predictive value; NPV, negative predictive value; PLR, positive likelihood ratio; NLR, negative likelihood ratio; CI, confidence.
